# Hemisynthesis and Biological Evaluation of Cinnamylated, Benzylated, and Prenylated Dihydrochalcones from a Common Bio-Sourced Precursor

**DOI:** 10.3390/antibiotics10060620

**Published:** 2021-05-22

**Authors:** Anne Ardaillou, Jérôme Alsarraf, Jean Legault, François Simard, André Pichette

**Affiliations:** Centre de Recherche sur la Boréalie (CREB), Laboratoire d’Analyse et de Séparation des Essences Végétales (LASEVE), Université du Québec à Chicoutimi, 555 Boulevard de l’Université, Chicoutimi, QC G7H 2B1, Canada; anne.ardaillou1@uqac.ca (A.A.); Jean_Legault@uqac.ca (J.L.); Francois1_Simard@uqac.ca (F.S.)

**Keywords:** antibacterial, Brønsted acid catalysis, dihydrochalcone, Friedel–Crafts alkylation, hemisynthesis, natural products, *Staphylococcus aureus*

## Abstract

Several families of naturally occurring *C*-alkylated dihydrochalcones display a broad range of biological activities, including antimicrobial and cytotoxic properties, depending on their alkylation sidechain. The catalytic Friedel–Crafts alkylation of the readily available aglycon moiety of neohesperidin dihydrochalcone was performed using cinnamyl, benzyl, and isoprenyl alcohols. This procedure provided a straightforward access to a series of derivatives that were structurally related to natural balsacones, uvaretin, and erioschalcones, respectively. The antibacterial and cytotoxic potential of these novel analogs was evaluated in vitro and highlighted some relations between the structure and the pharmacological properties of alkylated dihydrochalcones.

## 1. Introduction

**Figure 1:**
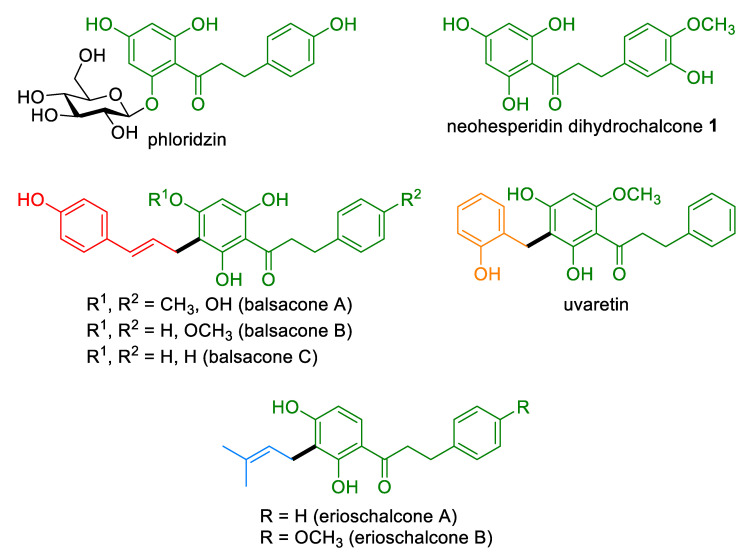
Structure of various dihydrochalcones, including the aglycone moiety of neohesperidin dihydrochalcone (**1**).

Dihydrochalcones (DHCs) are a class of natural phenylpropanoids derived from the biosynthetic pathway of shikimic acid [1]. They are the acyclic counterparts of flavanones and are found in several botanical families [2]. Their scaffold can feature various sidechains [3] resulting from *C*-glycosylation [4], *C*-cinnamylation [5,6,7,8], *C*-benzylation [9,10,11], or *C*-prenylation [12] (Figure 1). The structural diversity of DHCs translates into a broad range of biological activities such as antibacterial, antiviral, antidiabetic, antioxidant, anti-inflammatory, and cytotoxic properties [13]. Interestingly, DHCs may display different pharmacological properties compared to the corresponding chalcones. For instance, some recently described 4’-methyl DHCs were more active against *S. aureus* and less potent against *E. coli* than the corresponding chalcones [14]. While functionalized DHCs are commonly isolated in low yields, simpler derivatives can be found in high concentrations in plants. One of the most abundant natural DHCs is phloridzin, a DHC *O*-glucoside representing up to 14% of dry weight in apple tree leaves [15]. Some DHCs are also readily accessible by reduction of the corresponding chalcone or flavanone. This is for example the case of neohesperidin dihydrochalcone, a sweetener widely used in the food industry which is obtained by hydrogenation of the citrus metabolite neohesperidin [16]. We recently demonstrated that abundant DHCs can serve as precursors in the hemisynthesis of balsacones [17], a class of cinnamylated antibacterial and anti-psoriatic [18] DHCs from the bud of *Populus balsamifera* that are active against resistant strains of *S. aureus* (MRSA) and did not induce resistance in treated MRSA [19]. In this context, the use of natural DHCs advantageously saved the steps which would have been necessary for the construction of a DHC scaffold from petrochemicals. Their conversion into balsacones was performed in a single-step, following a metal-free and protective group-free Brønsted acid-catalyzed Friedel–Crafts alkylation protocol [20]. Bio-sourced cinnamyl alcohols were privileged as the alkylation reagents as their utilization saved a harmful halogenation step, and their conversion generated water as the lone byproduct making the process overall green [21,22]. The use of natural precursors was also profitable from a drug discovery perspective, as these renewable polyfunctional templates constitute a valuable source of structural diversity [23]. On the other hand, the scope of sidechains which could readily be incorporated onto the framework of natural DHCs remains to be explored to access the wider sidechain variety found in DHCs. 

Among these structures, *C*-benzylated DHCs have been found in the Annonaceae family, in particular in *Uvaria* species [9,10,24,25,26]. Uvaretin showed a considerable cytotoxicity against various human cancer cell lines with IC_50_ values in the low micromolar range [27]. Other 2-hydroxybenzyl-bearing flavonoids have also been found in nature such as the *C*-benzylated flavanone dichamanetin from *Piper sarmentosum*. Furthermore, *C*-prenylation is a relatively common modification in natural flavonoids that is considered to enhance their bioactivity by increasing lipophilicity [28,29]. *C*-prenylated and *C*-geranylated DHCs were notably found in the genus *Piper*. Recently, two prenylated DHCs, named erioschalcone A and B, were isolated in *Eriosema glomerata* and showed attractive antimicrobial properties against the pathogenic Gram-negative strains *Escherichia coli* and *Klebsiella pneumoniae* [12]. It is worthy of note that erioschalcones were inactive against *S. aureus*, unlike balsacones, which stressed the role of the alkylation fragment of DHCs on their antimicrobial properties. Herein, we devise the catalytic single-step *C*-cinnamylation, *C*-benzylation, and *C*-prenylation of the readily accessible neohesperidin dihydrochalcone aglycon **1** as a useful method to access derivatives that are structurally related to naturally occurring balsacones, uvaretins, and erioschalcones, respectively. The antibacterial and cytotoxic activity of the synthesized compound is also discussed. 

## 2. Results and Discussion

### 2.1. Chemical Synthesis

#### 2.1.1. Cinnamylation of DHC 1

Our first objective was to transpose the method developed for the hemisynthesis of balsacones to DHC **1** (Table 1). Briefly, the precursor **1** was treated with various cinnamyl alcohols **2a-e** in the presence of a catalytic amount of 4-toluenesulfonic acid (PTSA) in acetonitrile. Cinnamyl alcohols were prepared from their carboxylic acid counterparts following a straightforward esterification-reduction sequence [30]. 4-hydroxicinnamyl alcohol (**2a**) reacted smoothly with DHC **1** at room temperature, providing the expected product **3a** containing the same sidechain as natural balsacones in a good 69% yield (Table 1, entry 1). Analogues **3b** and **3c** were obtained from alcohols **2b** and **2c** in 49% and 61% yields, respectively (Table 1, entries 2–3). Cinnamyl alcohol (**2d**) was less reactive and required heating to produce 72% of the derivative **3d** (Table 1, entry 4). In the case of the chlorinated precursor **2e**, the intended product **3e** was obtained in a low 30% yield (Table 1, entry 5). This result was explained by the formation of several byproducts among which compound **3e’**, resulting from the alkylation of the trisubstituted aromatic ring of the DHC, was isolated in 9% yield (*see the Materials and methods section*). Nonetheless, a sufficient quantity of product **3e** was obtained for the biological assays.

#### 2.1.2. Benzylation of DHC 1

The next step of the study was the hemisynthesis of benzylated DHCs that would be structural analogs of uvaretin and other naturally occurring DHCs and flavanones. The total synthesis of uvaretin was reported in 1985 by Malterud et al. [31] and considerably improved very recently by Dallman et al. [27]. The seven-step route involved the construction of a protected chalcone framework, followed by a Friedel–Crafts alkylation using a protected 2-hydroxybenzyl precursor prior to the removal of all the protective-groups, providing uvaretin in a 15% overall yield. In this multistep strategy, the use of protective groups resulted in a 72% carbon economy (CE) in the final deprotection alone. In sharp contrast, our goal was to perform the direct alkylation of unprotected DHC **1** with 2-hydroxybenzyl alcohol **4a**, providing a *C*-benzylated DCH in a single step and with a 100% CE (Table 2). Pleasingly, DHC **1** and benzyl alcohol **4a** reacted cleanly in acetonitrile with a catalytic amount of PTSA, affording the alkylation product **5a** featuring the same 2-hydroxybenzyl fragment as uvaretin in a good 61% yield (Table 2, entry 1). It is worthy of note that the solvent did not seem to interfere with the reaction, as no traces of acetamide that would result from a Ritter reaction were observed [32]. The benzylation of DHC **1** was further extended to various substituted precursors, providing several analogues **5b–d** in yields ranging from 36% to 74% (Table 2, entries 2–4).

#### 2.1.3. Prenylation of DHC **1**

Finally, we envisioned the preparation of a *C*-prenylated derivative from DHC **1**. The single synthesis of a prenylated DHC that we were able to retrieve was a chemoenzymatic transformation of phloretin involving dimethylallyltryptophan synthase that afforded a *C*-prenylated counterpart in a modest 14.5% yield [33]. On the other hand, several syntheses of various *C*-prenylated flavonoids and phloroglucinol derivatives have been reported. They mainly relied on the alkylation of acylphloroglucinol scaffolds using allylbromides [34,35], and only a few studies involved prenol (**6a**) or 2-methyl-3-buten-2-ol (**6b**) as the alkylating agent. A representative example was xanthohumol. In the total synthesis [36] of this *C*-prenylated chalcone from hops (*Humulus lupulus* L.), prenol (**6a**) reacted with a protected acetophloroglucinol derivative under Mitsunobu conditions to provide an ether. A Claisen rearrangement then occurred upon heating to yield a *C*-prenylated intermediate. The chalcone scaffold was then constructed following a Claisen–Schmidt condensation with a protected aldehyde prior to a final deprotection. Overall, the total synthesis of xanthohumol required six steps from acetophloroglucinol with an 11% overall yield. Once again, an alternative method based on the direct prenylation of a natural DHC would be highly desirable in both terms of step- and atom-economy.

The reaction between DHC (**1**) and 1 equivalent of 3-methyl-2-buten-1-ol (**6a**) in the presence of a catalytic amount of PTSA afforded a mixture of the expected *C*-alkylation product **7a**, featuring the same alkylation motif as erioschalcones, along with an *O*-alkylation byproduct **7b** with a 40% yield (Table 3, entry 1). This moderate yield was slightly improved to 48% when 0.50 equivalent of alcohol **6a** was used (Table 3, entry 2). Pleasingly, replacing the prenol **6a** with its isomer 2-methyl-3-buten-2-ol **6b** increased the yield to 58% (Table 3, entry 4). These results were considered as satisfactory as our single-step protocol was the first hemisynthesis of a *C*-prenylated DHC and provided a better yield than the conventional multistep procedures reported for the synthesis of related *C*-prenylated phenylpropanoids. Careful separation by reverse-phase chromatography provided pure samples of the two prenylation products **7a** and **7b** for biological assays. As *O*-prenylation is a modification found in antimicrobial natural flavonoids and acetophloroglucinols [37], the *O*-prenylated DHC **7b** was also subjected to biological assays.

**Scheme 1:**
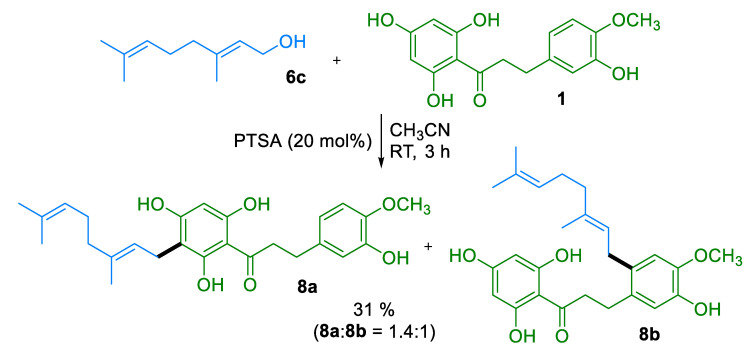
Catalytic Geranylation of Dihydrochalcone **1**.

Extension of these experimental conditions to geraniol **6c** (Scheme 1) led to a mixture of the *C*-geranylated DHC **8a** with a byproduct **8b** in a modest 31% yield. Yet, careful separation of the *C*-geranylated DHC **8a** by reverse-phase chromatography enabled its isolation in sufficient quantity to carry out the evaluation of its biological properties.

### 2.2. Biological Evaluation of Alkylated DHCs **3a–e, 5a–d, 7a–b,** and **8a**

The antibacterial and cytotoxic activities of derivatives **3a**–**e**, **5a**–**d**, **7a**–**b**, and **8a** were evaluated in vitro and compiled in Table 4. The results of antibacterial assays, performed against Gram-negative *Escherichia coli* (ATCC 25922) and Gram-positive *Staphylococcus aureus* (ATCC 25923), were expressed as the minimal concentration inhibiting 90% of bacterial growth (MIC_90_) using gentamicin and balsacone A as positive controls. The cytotoxicity against two malignant (A-549 and DLD-1) and one healthy (WS-1) human cell lines was reported as the half maximal inhibitory concentration (IC_50_) using etoposide as a positive control. 

The cinnamylated DHCs **3a-e** displayed significant inhibitory properties against *S. aureus*, with MIC_90_ values ranging from 2.6 to 11.6 µM (Table 4, entries 1–5). They were inactive against *E. coli*, which was consistent with the recorded antibacterial potential of the naturally occurring cinnamylated DHC balsacone A [5] (Table 4, entry 12). They also confirmed our previous hypothesis that the substitution of the DHC scaffold had a limited impact on the antibacterial potential of cinnamylated DHCs [17]. Notably, the derivative **3a** (Table 4, entry 1), featuring the same 4-hydroxycinnamyl fragment as balsacone A, was the least active among the five new compounds **3a-e**. On the other hand, the chlorinated counterpart **3e** (Table 4, entry 5) was slightly more active than balsacone A, which suggested that modulating the nature of the alkylation motif was a relevant approach to modulate the antibacterial properties of cinnamylated DHCs. The novel benzylated DHCs devised here were not cytotoxic against the three tested lines. These results contrasted with the reported activity of the natural *C*-benzylated DHC uvaretin that inhibited the growth of A549 cells with a 2.2 µM IC_50_ [27]. Therefore, the substitution pattern of benzylated DHCs played a pivotal role towards their cytotoxic activity. On the other hand, benzylated compounds **5a–d** were active against *S. aureus* (Table 4, entries 6–9). Once again, the chlorinated derivative was the most active of the array (Table 4, entry 9) and surpassed balsacone A. Strikingly, the *C*-prenylated DHC **7a** showed a marginal inhibition of *E. coli* (Table 4, entry 10), which did not match the reported activity of natural erioschalcones [12]. While the substitution of the DHC scaffold had a limited impact on the antimicrobial properties of cinnamylated DHCs, this result suggested that the substitution pattern of prenylated DHCs was an important feature towards their activity against *E. coli*. On the other hand, the geranylated DHC **8a** was slightly more active than balsacone A against *S. aureus* (Table 4, entry 12). Further efforts towards the direct prenylation and geranylation of other naturally occurring DHCs could therefore highlight the structural features responsible for the antibacterial properties of this class of natural products.

## 3. Materials and Methods

### 3.1. General Information

#### 3.1.1. Chemistry

Unless otherwise noted, all starting materials and solvents were purchased from commercial sources (Sigma-Aldrich, Acros Organics) and used as received without further purification. Reactions were conducted under argon atmosphere, using anhydrous solvent, unless otherwise noted. All reactions were monitored by thin-layer chromatography (TLC) using normal phase silica gel 60 F_254_ 0.25 mm or reverse phase silica gel 60 RP-18 F_254_s 0.25 mm precoated aluminum foil plates (Silicycle). Normal phase TLC were visualized under UV (254 nm) or revealed using H_2_SO_4_ under UV (365 nm), as for reverse phase TLC. Flash chromatographic purifications were performed using normal phase silica gel 60 (15–40 μm) or C-18 reverse-phase silica gel (17%) (40–63 μm) columns. NMR spectra were recorded with a Bruker Avance 400 spectrometer at 400 MHz for ^1^H nuclei and 101 MHz for ^13^C nuclei, using deuterated acetonitrile or methanol as the solvent. Chemical shifts δ were reported in ppm relative to the solvent residual peak (CD_3_OD δ_H/C_ 3.31/49.00 ppm; CD_3_CN δ_H/C_ 1.94/118.26 ppm; CDCl_3_ δ_H__/__C_ 7.26/77.16 ppm) [38] and coupling constants *J* in Hertz (Hz). Copies of ^1^H and ^13^C NMR spectra of new compounds are given in the Supplementary Materials. HRMS spectra were recorded on an Agilent 6224 MS-TOF mass spectrometer equipped with an electrospray source.

Cinnamyl alcohols were prepared from the corresponding acids following a known procedure [30]. DHC **1** was prepared from neohesperidin dihydrochalcone according to a known procedure [39].

#### 3.1.2. Antibacterial Assays

Gram-negative *Escherichia coli* (ATCC 25922) and Gram-positive *Staphylococcus aureus* (ATCC 25923) were obtained from the American Type Culture Collection (ATCC). Antibacterial assays were performed following a modified microdilution method [40]. Briefly, exponentially growing bacteria (density of 5 × 10^3^ *E. coli* or 3.5 × 10^4^ *S. aureus*) were plated in 96-well round-bottom microplates per well in 100 μL of nutrient broth. The tested compounds were solubilized in DMSO and then diluted in nutrient broth or Sabouraud dextrose so that the final concentration of DMSO was maintained below 0.1% (*v*/*v*) to avoid solvent toxicity. Bacteria were treated using increasing concentrations of tested compounds and incubated for 24 h at 37 °C. Absorbance was read using a Varioskan Ascent plate reader (Thermo Electron) at 600 nm and the results were expressed as the concentration inhibiting 90% of bacterial growth (MIC_90_).

#### 3.1.3. Cytotoxicity Assays

A-549 (lung cancer, ATCC # CCL-185), DLD-1 (colon cancer, ATCC # CCL-221) and WS-1 (skin fibroblasts, ATCC # CRL-1502) cell lines were obtained from the American Type Culture Collection (ATCC, Manassas, VA, USA). Cell lines were grown. Cells were cultured at 37 °C in a humidified atmosphere containing 5% CO_2_ in minimum essential medium containing Earle’s salt (Mediatech Cellgro, Herndon, VA, USA), supplemented with 10% fetal calf serum (Hyclone, Logan, UT, USA), 1 × solution of vitamins, 1 × sodium pyruvate, 1 × non-essential amino acids, 60 µg/mL of penicillin sodium, and 100 µg/mL of streptomycin (Mediatech Cellgro). Exponentially growing cells were plated at a density of 5 × 10^3^ cells per well in 96-well microplates (BD Falcon) in culture medium (100 µL). Cells were allowed to adhere for 16 h before treatment. The tested compounds were solubilized in DMSO and then diluted in cell culture medium so that the final concentration of DMSO was maintained below 0.5% (*v*/*v*) to avoid solvent toxicity. Cells were treated using increasing concentrations of tested compounds and incubated for 48 h at 37 °C. Cytotoxicity was assessed using Hoechst 33342 (bisbenzimide) fluorometric assay [41]. Briefly, after removal of the supernatant, cells were incubated at room temperature during 1 h with sodium dodecyl sulfate (100 µL of 0.02% solution in sterile distilled water) and then frozen overnight. After thawing, a solution of Hoechst 33342 dye in TNE 2x (10 mM tris-HCl, pH 7.4, 1 mM EDTA, 4 M NaCl) was added to reach a final concentration of 30 µg/mL. The plates were incubated at room temperature during 2 h in the dark and fluorescence was measured at 355/460 nm (excitation/emission) using a microplate fluorescence reader. The results were expressed as the concentration of drug inhibiting cell growth by 50% (IC_50_).

### 3.2. Synthesis of Compounds **3a–e**

#### 3.2.1. General Procedure for the Cinnamylation of DHC **1**

A solution of 4-toluenesulfonic acid monohydrate (catalytic loading indicated in Table 1) in acetonitrile (0.1 mL) was added to a solution of DHC **1** (0.40 mmol) and cinnamyl alcohol **2** (0.10 mmol) in acetonitrile (1.9 mL or 2.9 mL). The mixture was stirred at the temperature and for the time indicated in Table 1. After dilution with 5 mL ethyl acetate, the mixture was quenched with a saturated aqueous solution of NaHCO_3_ (20 mL) and extracted with ethyl acetate (3 × 20 mL). The organic layers were combined, dried over Na_2_SO_4_, filtered, and the solvent was rotary evaporated. The expected cinnamylation product **3** was finally obtained after flash chromatography on reverse-phase (C18) silica gel using a gradient from 50% to 90% of methanol in water as the eluent. 

#### 3.2.2. Compound **3a**

(*E*)-3-(3-hydroxy-4-methoxyphenyl)-1-(2,4,6-trihydroxy-3-(3-(4-hydroxyphenyl)allyl)phenyl)propan-1-one. Following the general procedure above, a mixture of DHC **1** (0.120 g, 0.395 mmol) and 4-hydroxycinnamic alcohol (**2a**) (0.0147 g, 0.0979 mmol) afforded the title compound **3a** as a pale orange powder (29.5 mg, 69%). *R_f_* = 0.60 (CHCl_3_/MeOH 9:1); HRMS (ESI) m/z calcd for C_25_H_24_O_7_ [M − H]^–^ 437.1595, found 437.1594; ^1^H NMR (400 MHz, CD_3_OD): δ 7,13 (d, *J* = 8.5 Hz, 2H), 6.79 (d, *J* = 8.2 Hz, 1H), 6.71 (d, *J* = 1.7 Hz, 1H), 6.69 – 6.62 (m, 3H), 6.26 (d, *J* = 15.8 Hz, 1H), 6.11 (dt, *J* = 6.4, 15.5 Hz, 1H), 5.95 (s, 1H), 3.79 (s, 3H), 3.36 (d, *J* = 6.2 Hz, 2H), 3.28 (t, *J* = 8.2 Hz, 2H), 2.83 (t, *J* = 8.1 Hz, 2H); ^13^C NMR (101 MHz, CD_3_OD): δ 206.4, 165.1, 163.9, 161.7, 157.3, 147.3, 147.2, 136.2, 131.3, 130.1, 128.0, 126.8, 120.5, 116.5, 116.1, 112.8, 106.6, 105.2, 94.9, 56.5, 47.2, 31.7, 26.4. 

#### 3.2.3. Compound **3b**

(*E*)-3-(3-hydroxy-4-methoxyphenyl)-1-(2,4,6-trihydroxy-3-(3-(4-methoxyphenyl)allyl)phenyl)propan-1-one. Following the general procedure above, a mixture of DHC **1** (0.130 g, 0.432 mmol) and 4-methoxycinnamic alcohol (**2b**) (0.0178 g, 0.108 mmol) afforded the title compound **3b** as a yellow powder (23.8 mg, 49%). *R_f_* = 0.46 (CHCl_3_/MeOH 9:1); HRMS (ESI) m/z calcd for C_26_H_26_O_7_ [M − H]^–^ 451.1751, found 451.1753; ^1^H NMR (400 MHz, CD_3_OD): δ 7.20 (d, *J* = 8.7 Hz, 2H), 6.83 – 6.75 (m, 3H), 6.71 (d, *J* = 1.8 Hz, 1H), 6.65 (dd, *J* = 1.8, 8.1 Hz, 1H), 6.28 (d, *J* = 15.8 Hz, 1H), 6.16 (dt, *J* = 6.4, 15.9 Hz, 1H), 5.95 (s, 1H), 3.79 (s, 3H), 3.73 (s, 3H), 3.37 (d, *J* = 6.0 Hz, 2H), 3.28 (t, *J* = 8.2 Hz, 2H), 2.83 (t, *J* = 7.5 Hz, 2H); ^13^C NMR (101 MHz, CD_3_OD): δ 206.4, 165.1, 163.9, 161.8, 160.0, 147.3, 147.2, 136.2, 132.3, 129.9, 128.0, 127.6, 120.5, 116.5, 114.8, 112.9, 106.5, 105.2, 94.9, 56.5, 55.6, 47.2, 31.7, 26.4. 

#### 3.2.4. Compound **3c**

(*E*)-1-(3-(3-(3,4-dimethoxyphenyl)allyl)-2,4,6-trihydroxyphenyl)-3-(3-hydroxy-4-methoxyphenyl)propan-1-one. Following the general procedure above, a mixture of DHC **1** (0.125 g, 0.412 mmol) and 3,4-dimethoxycinnamic alcohol (**2c**) (0.0201 g, 0.104 mmol) afforded the title compound **3c** as a white powder (30.6 mg, 61%). *R_f_* = 0.50 (CHCl_3_/MeOH 9:1); HRMS (ESI) m/z calcd for C_27_H_28_O_8_ [M − H]^–^ 481.1857, found 481.1859; ^1^H NMR (400 MHz, CD_3_OD): δ 13.81 (br s, 1H), 8.04 (br s, 1H), 6.92 (s, 1H), 6.86 – 6.78 (m, 3H), 6.72 (d, *J* = 1.9 Hz, 1H), 6.67 (dd, *J* = 1.9, 8.2 Hz, 1H), 6.26 (d, *J* = 15.9 Hz, 1H), 6.19 (dt, *J* = 5.8, 15.8 Hz, 1H), 5.98 (s, 1H), 3.80 (s, 3H), 3.77 (s, 3H), 3.75 (s, 3H), 3.36 (d, *J* = 5.7 Hz, 2H), 3.28 (t, *J* = 8.2 Hz, 2H), 2.82 (t, *J* = 7.5 Hz, 2H); ^13^C NMR (101 MHz, CD_3_OD): δ 206.0, 164.9, 162.4, 160.6, 150.1, 149.3, 146.8, 146.4, 135.9, 131.8, 130.0, 127.2, 120.4, 119.6, 115.9, 112.6, 112.5, 109.6, 106.2, 105.1, 94.9, 56.6, 56.2, 56.1, 46.7, 30.6, 26.0.

#### 3.2.5. Compound **3d**

1-(3-cinnamyl-2,4,6-trihydroxyphenyl)-3-(3-hydroxy-4-methoxyphenyl)propan-1-one. Following the general procedure above, a mixture of DHC **1** (0.122 g, 0.40 mmol) and cinnamic alcohol (**2d**) (0.0134 g, 0.10 mmol) afforded the title compound **3d** as a white powder (30.1 mg, 72%). *R_f_* = 0.63 (CHCl_3_/MeOH 9:1); HRMS (ESI) m/z calcd for C_25_H_24_O_6_ [M − H]^–^ 421.1646, found 421.1653; ^1^H NMR (400 MHz, CD_3_OD): δ 7.28 (d, *J* = 7.5 Hz, 2H), 7.21 (t, *J* = 7.2 Hz, 2H), 7.11 (t, *J* = 7.2 Hz, 1H), 6.79 (d, *J* = 8.1 Hz, 1H), 6.71 (s, 1H), 6.65 (d, *J* = 8.1 Hz, 1H), 6.40 – 6.24 (m, 2H), 5.96 (s, 1H), 3.79 (s, 3H), 3.40 (d, *J* = 3.4 Hz, 2H), 3.29 (t, *J* = 8.1 Hz, 2H), 2.83 (t, *J* = 7.9 Hz, 2H); ^13^C NMR (101 MHz, CD_3_OD): δ 206.4, 165.2, 163.9, 161.8, 147.3, 147.2, 139.5, 136.2, 130.4, 129.8, 129.4, 127.5, 126.9, 120.5, 116.5, 112.8, 106.2, 105.2, 94.9, 56.5, 47.2, 31.7, 26.5.

#### 3.2.6. Compound **3e**

(*E*)-1-(3-(3-(4-chlorophenyl)allyl)-2,4,6-trihydroxyphenyl)-3-(3-hydroxy-4-methoxyphenyl)propan-1-one. Following the general procedure above, a mixture of DHC **1** (0.0656 g, 0.216 mmol) and 4-chlorocinnamic alcohol (**2e**) (0.0184 g, 0.108 mmol) afforded the title compound **3e** as a white powder (13.1 mg, 30%) and compound **3e’** as a white powder (4.2 mg, 9%). *R_f_* = 0.40 (CHCl_3_/MeOH 9:1); HRMS (ESI) m/z calcd for C_25_H_23_ClO_6_ [M − H]^–^ 455.1255, found 455.1256; ^1^H NMR (400 MHz, CD_3_OD): δ 7.26 (d, *J* = 8.6 Hz, 2H), 7.22 (s, 1H), 7.19 (d, *J* = 7.0 Hz, 1H), 6.79 (d, *J* = 8.2 Hz, 1H), 6.71 (d, *J* = 1.8 Hz, 1H), 6.65 (dd, *J* = 1.9, 8.2 Hz, 1H), 6.39 – 6.25 (m, 2H), 5.96 (s, 1H), 3.79 (s, 3H), 3.39 (d, *J* = 4.2 Hz, 2H), 3.28 (t, *J* = 8.2 Hz, 2H), 2.82 (t, *J* = 8.1 Hz, 2H); ^13^C NMR (101 MHz, CD_3_OD): δ 206.5, 164.0, 162.0, 147.2, 138.3, 136.2, 133.0, 130.9, 130.1, 129.4, 129.1, 128.6, 128.3, 120.6, 116.5, 112.9, 106.0, 105.2, 95.0, 56.5, 47.1, 31.7, 26.4.

#### 3.2.7. Compound **3e’**

(*E*)-3-(2-(3-(4-chlorophenyl)allyl)-5-hydroxy-4-methoxyphenyl)-1-(2,4,6-trihydroxyphenyl)propan-1-one.

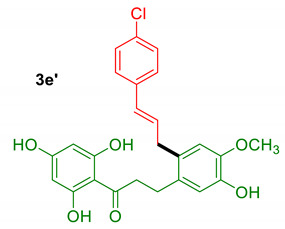



*R_f_* = 0,40 (CHCl_3_/MeOH 9/1); ^1^H NMR (400 MHz, CD_3_OD): δ 7.27 (d, *J* = 8.5 Hz, 2H), 7.20 (d, *J* = 8.6 Hz, 2H), 6.74 (s, 1H), 6.70 (s, 1H), 6.44 – 6.29 (m, 2H), 5.79 (s, 2H), 3.81 (s, 3H), 3.50 (d, *J* = 5.4 Hz, 2H), 3.25 (t, *J* = 8.3 Hz, 2H), 2.87 (t, *J* = 8.1 Hz, 2H); ^13^C NMR (101 MHz, CD_3_OD): δ 206.2, 166.3, 165.8, 147.3, 146.0, 137.8, 133.9, 133.4, 132.0, 130.3, 129.9, 129.5, 128.4, 117.5, 114.7, 105.3, 95.8, 56.5, 46.6, 37.0, 28.5.

### 3.3. Synthesis of Compounds **5a–d**

#### 3.3.1. General Procedure for the Benzylation of DHC **1**

A solution of 4-toluenesulfonic acid monohydrate (catalytic loading indicated in Table 2) in acetonitrile (0.1 mL) was added to a solution of DHC **1** (0.20 mmol) and benzyl alcohol **4** (0.10 mmol) in acetonitrile (1.9 mL or 2.9 mL). The mixture was stirred at room temperature (unless otherwise noted in Table 1) for 2 or 3 h. The mixture was then diluted with ethyl acetate (5 mL), quenched with a saturated aqueous solution of NaHCO_3_ (20 mL) and extracted three times with ethyl acetate (3 × 20 mL). The organic layers were pooled together, dried over Na_2_SO_4_, filtered, and the solvent was rotary evaporated. The crude residue was chromatographed over reverse-phase (C-18) silica gel using a gradient of methanol in water (from 50% to 90%) as the mobile phase to afford the expected benzylation product **5**.

#### 3.3.2. Compound **5a**

3-(3-hydroxy-4-methoxyphenyl)-1-(2,4,6-trihydroxy-3-(2-hydroxybenzyl)phenyl)propan-1-one. Following the general procedure above, a mixture of DHC **1** (0.0602 g, 0.20 mmol) and 2-hydroxybenzyl alcohol (**4a**) (0.0125 g, 0.10 mmol) afforded the title compound **5a** as a yellow powder (25.0 mg, 61%). *R_f_* = 0.40 (CHCl_3_/MeOH 9:1); HRMS (ESI) m/z calcd for C_23_H_22_O_7_ [M − H]^–^ 411.1435, found 411.1438; ^1^H NMR (400 MHz, CD_3_OD): δ 7.21 (d, *J* = 7.5 Hz, 1H), 7.03 (t, *J* = 7.0 Hz, 1H), 6.84 – 6.75 (m, 3H), 6.72 (d, *J* = 1.7 Hz, 1H), 6.66 (dd, *J* = 1.5, 8.1 Hz, 1H), 5.99 (s, 1H), 3.80 (s, 3H), 3.76 (s, 2H), 3.28 (t, *J* = 7.9 Hz, 2H), 2.81 (t, *J* = 7.6 Hz, 2H); ^13^C NMR (101 MHz, CD_3_OD): δ 206.3, 163.9, 162.4, 161.2, 154.6, 146.8, 146.3, 135.8, 131.5, 128.2, 127.8, 121.1, 120.4, 116.1, 115.8, 112.5, 107.0, 105.1, 95.5, 56.6, 46.6, 30.6, 22.8.

#### 3.3.3. Compound **5b**

3-(3-hydroxy-4-methoxyphenyl)-1-(2,4,6-trihydroxy-3-(4-hydroxybenzyl)phenyl)propan-1-one. Following the general procedure above, a mixture of DHC **1** (0.0608 g, 0.20 mmol) and 4-hydroxybenzyl alcohol (**4b**) (0.0125 g, 0.10 mmol) afforded the title compound **5b** as a yellow powder (30.3 mg, 74%). *R_f_* = 0.30 (CHCl_3_/MeOH 9:1); HRMS (ESI) m/z calcd for C_23_H_22_O_7_ [M − H]^–^ 411.1437, found 411.1438; ^1^H NMR (400 MHz, CD_3_OD): δ 7.07 (d, *J* = 8.4 Hz, 2H), 6.79 (d, *J* = 8.2 Hz, 1H); 6.71 (d, *J* = 1.8 Hz, 1H), 6.68 – 6.57 (m, 3H), 5.94 (s, 1H), 3.80 (s, 3H), 3.73, (s, 2H), 3.28 (t, *J* = 8.3 Hz, 2H), 2.82 (t, *J* = 7.7 Hz, 2H); ^13^C NMR (101 MHz, CD_3_OD): δ 206.4, 165.2, 163.9, 161.7, 155.8, 147.3, 147.2, 136.2, 134.4, 130.4, 120.5, 116.5, 115.6, 112.9, 108.5, 105.2, 94.9, 56.5, 47.2, 31.8, 27.8.

#### 3.3.4. Compound **5c**

3-(3-hydroxy-4-methoxyphenyl)-1-(2,4,6-trihydroxy-3-(4-methoxybenzyl)phenyl)propan-1-one. Following the general procedure above, a mixture of DHC **1** (0.129 g, 0.426 mmol) and 4-methoxybenzyl alcohol (**4c**) (0.0147 g, 0.106 mmol) afforded the title compound **5c** as a white powder (16.4 mg, 36%). *R_f_* = 0.46 (CHCl_3_/MeOH 9:1); HRMS (ESI) m/z calcd for C_24_H_24_O_7_ [M − H]^–^ 425.1595, found 425.1586; ^1^H NMR (400 MHz, CD_3_OD): δ 7.12 (d, *J* = 8.5 Hz, 2H), 6.74 (d, *J* = 8.2 Hz, 1H), 6.69 (d, *J* = 8.6 Hz, 2H), 6.67 (d, *J* = 1.8 Hz, 1H), 6.61 (dd, *J* = 1.6, 8.3 Hz, 1H), 5.90 (s, 1H), 3.75 (s, 3H), 3.73 (s, 2H), 3.67 (s, 3H), 3.24 (t, *J* = 8.2 Hz, 2H), 2.78 (t, *J* = 7.6 Hz, 2H); ^13^C NMR (101 MHz, CD_3_OD): δ 206.4, 165.2, 163.9, 161.7, 158.9, 147.3, 147.2, 136.2, 135.5, 130.4, 120.5, 116.5, 114.2, 112.8, 108.3, 105.2, 94.8, 56.5, 55.6, 47.2, 31.7, 27.8.

#### 3.3.5. Compound **5d**

1-(3-(4-chlorobenzyl)-2,4,6-trihydroxyphenyl)-3-(3-hydroxy-4-methoxyphenyl)propan-1-one. Following the general procedure above, a mixture of DHC **1** (0.0618 g, 0.20 mmol) and 4-chlorobenzyl alcohol (**4d**) (0.0143 g, 0.10 mmol) afforded the title compound **5d** as a white powder (12.8 mg, 30%). *R_f_* = 0.57; ^1^H NMR (400 MHz, CD_3_OD): δ 7.31 (d, *J* = 8.3 Hz, 2H), 7.17 (d, *J* = 8.3 Hz, 2H), 6.79 (d, *J* = 8.2 Hz, 1H), 6.70 (d, *J* = 1.7 Hz, 1H), 6.65 (dd, *J* = 1.6, 8.1 Hz, 1H), 5.95 (s, 1H), 3.81 (s, 3H), 3.79 (s, 2H), 3.28 (t, *J* = 8.1 Hz, 2H), 2.82 (t, *J* = 8.1 Hz, 2H); ^13^C NMR (101 MHz, CD_3_OD): δ 206.4, 163.9, 162.0, 147.3, 147.2, 142.8, 136.2, 131.8, 131.6, 120.5, 119.8, 116.5, 112.9, 111.4, 107.3, 105.2, 94.8, 56.5, 47.2, 31.7, 28.2.

### 3.4. Synthesis of Compounds **7a–b** and **8a–b**

#### 3.4.1. Prenylation of DHC **1**

A solution of 4-toluenesulfonic acid monohydrate (catalytic loading indicated in Table 3) in acetonitrile (0.1 mL) was added to a solution of DHC **1** (0.121 g, 0.40 mmol) and prenol **6a** (10.2 µL, 0.10 mmol) in acetonitrile (1.9 mL). The mixture was stirred at room temperature for 2 h. The mixture was then diluted with ethyl acetate (5 mL), quenched with a saturated aqueous solution of NaHCO_3_ (20 mL) and extracted three times with ethyl acetate (3 × 20 mL). The organic layers were pooled together, dried over Na_2_SO_4_, filtered, and the solvent was rotary evaporated. The crude residue was chromatographed over reverse-phase (C-18) silica gel using a gradient of methanol in water (from 50% to 90%) as the mobile phase to afford a mixture of compounds **7a** and **7b** as a yellow powder (16.5 mg, 48%) with a ratio of 1.3:1 (**7a**:**7b**). Pure samples of **7a** and **7b** were obtained by thorough separation by chromatography over reverse-phase (C-18) silica gel. 

#### 3.4.2. Compound **7a**

3-(3-hydroxy-4-methoxyphenyl)-1-(2,4,6-trihydroxy-3-(3-methylbut-2-en-1-yl)phenyl)propan-1-one. *R_f_* = 0.50 (CHCl_3_/MeOH 9:1); HRMS (ESI) m/z calcd for C_21_H_24_O_6_ [M − H]^–^ 373.1639, found 373.1646; ^1^H NMR (400 MHz, CD_3_OD): δ 6.80 (d, *J* = 8.2 Hz, 1H), 6.71 (d, *J* = 1.8 Hz, 1H), 6.65 (dd, *J* = 2.0, 8.3 Hz, 1H), 5.90 (s, 1H), 5.17 (t, *J* = 6.9 Hz, 1H), 3.80 (s, 3H), 3.27 (t, *J* = 8.2 Hz, 2H), 3.18 (d, *J* = 6.7 Hz, 2H), 2.82 (t, *J* = 8.1 Hz, 2H), 1.74 (s, 3H), 1.64 (s, 3H); ^13^C NMR (101 MHz, CD_3_OD): δ 205.1, 161.0, 145.4, 144.9, 135.7, 135.1, 121.8, 120.1, 114.8, 114.6, 110.8, 105.9, 104.9, 95.3, 56.1, 46.0, 30.3, 25.9, 21.7, 18.0.

#### 3.4.3. Compound **7b**

3-(4-methoxy-3-((3-methylbut-2-en-1-yl)oxy)phenyl)-1-(2,4,6-trihydroxyphenyl)propan-1-one. *R_f_* = 0.50 (CHCl_3_/MeOH 9:1); HRMS (ESI) m/z calcd for C_21_H_24_O_6_ [M − H]^–^ 373.1640, found 373.1646; ^1^H NMR (400 MHz, CD_3_OD): δ 6.81 (d, *J* = 8.1 Hz, 1H), 6.71 (s, 1H), 6.66 (d, *J* = 8.2 Hz, 1H), 5.81 (s, 2H), 5.19 (t, *J* = 6.5 Hz, 1H), 3.81 (s, 3H), 3.74 (d, *J* = 6.9 Hz, 2H), 3.27 (t, *J* = 8.0 Hz, 2H), 2.82 (t, *J* = 8.0 Hz, 2H), 1.72 (s, 3H), 1.68 (s, 3H); ^13^C NMR (101 MHz, CD_3_OD): δ 206.2, 166.4, 165.9, 147.3, 147.2, 136.7, 136.2, 121.3, 120.5, 116.5, 112.9, 111.4, 105.2, 95.8, 56.5, 47.1, 38.4, 31.6, 25.8, 22.4, 17.8.

#### 3.4.4. Geranylation of DHC **1**

A solution of 4-toluenesulfonic acid monohydrate (catalytic loading indicated in Table 3) in acetonitrile (0.1 mL) was added to a solution of DHC **1** (0.1123 g, 0.37 mmol) and geraniol **6c** (16.7 µL, 0.092 mmol) in acetonitrile (1.9 mL) was added. The mixture was stirred at room temperature for 2 h. The mixture was then diluted with ethyl acetate (5 mL), quenched with a saturated aqueous solution of NaHCO_3_ (20 mL) and extracted three times with ethyl acetate (3 × 20 mL). The organic layers were pooled together, dried over Na_2_SO_4_, filtered, and the solvent was rotary evaporated. The crude residue was chromatographed over reverse-phase (C-18) silica gel using a gradient of methanol in water (from 50% to 90%) as the mobile phase to afford a mixture of compounds **8a** and **8b** as a white powder (13.3 mg, 31%) with a ratio of 1.4:1 (**8a**:**8b**). Pure samples of **8a** and **8b** were obtained by thorough separation by chromatography over reverse-phase (C-18) silica gel.

#### 3.4.5. Compound **8a**

(*E*)-1-(3-(3,7-dimethylocta-2,6-dien-1-yl)-2,4,6-trihydroxyphenyl)-3-(3-hydroxy-4-methoxyphenyl)propan-1-one. *R_f_* = 0.56 (CHCl_3_/MeOH 9:1); HRMS (ESI) m/z calcd for C_26_H_32_O_6_ [M − H]^–^ 441.2271, found 441.2272; ^1^H NMR (400 MHz, CD_3_OD): δ 8.16 (br s, 1H), 7.61 (br s, 1H), 6.82 (d, *J* = 8.1 Hz, 1H), 6.71 (s, 1H), 6.67 (d, *J* = 7.6 Hz, 1H), 6.41 (br s, 1H), 5.95 (s, 1H), 5.13 (t, *J* = 6.5 Hz, 1H), 5.05 (t, *J* = 5.4 Hz, 1H), 3.80 (s, 3H), 3.26 (t, *J* = 7.8 Hz, 2H), 3.17 (d, *J* = 6.5 Hz, 2H), 2.81 (t, *J* = 7.4 Hz, 2H), 2.30 (br s, 1H), 2.23 – 1.90 (m, 3H), 1.72 (s, 3H), 1.61 (s, 3H), 1.55 (s, 3H); ^13^C NMR (101 MHz, CD_3_OD): δ 205.9, 164.7, 162.2, 160.2, 146.8, 146.4, 135.9, 135.6, 132.1, 125.2, 123.7, 120.5, 115.9, 112.6, 108.0, 105.1, 95.0, 56.7, 46.7, 40.3, 30.7, 27.3, 25.7, 21.8, 17.7, 16.2.

#### 3.4.6. Compound **8b**

(*E*)-1-(3-(3,7-dimethylocta-2,6-dien-1-yl)-2,4,6-trihydroxyphenyl)-3-(3-hydroxy-4-methoxyphenyl)propan-1-one. *R_f_* = 0.56 (CHCl_3_/MeOH 9:1); HRMS (ESI) m/z calcd for C_26_H_32_O_6_ [M − H]^–^ 441.2269, found 441.2272; ^1^H NMR (400 MHz, CD_3_OD): δ 10.90 (br s, 2H), 7.71 (br s, 1H), 6.73 (s, 1H), 6.64 (s, 1H), 6.25 (br s, 1H), 5.85 (s, 2H), 5.19 (t, *J* = 7.0 Hz, 1H), 5.07 (t, *J* = 6.8 Hz, 1H), 3.80 (s, 3H), 3.26 (d, *J* = 6.9 Hz, 2H), 3.19 (t, *J* = 8.2 Hz, 2H), 2.80 (t, *J* = 8.2 Hz, 2H), 2.04 (t, *J* = 7.4 Hz, 2H), 1.98 (d, *J* = 7.6 Hz, 2H) 1.68 (s, 3H), 1.62 (s, 3H), 1.55 (s, 3H); ^13^C NMR (101 MHz, CD_3_OD): δ 205.9, 165.1, 164.7, 146.3, 145.1, 136.2, 133.2, 132.2, 131.8, 125.1, 124.7, 116.6, 114.0, 110.9, 105.2, 95.8, 56.7, 46.2, 40.3, 31.9, 27.5, 27.3, 25.7, 17.7, 16.3.

## 4. Conclusions

In summary, we devised the single-step hemisynthesis of *C*-cinnamylated, *C*-benzylated, and *C*-prenylated DHCs from the readily accessible aglycon moiety of neohesperidin dihydrochalcone **1**. This protective group-free approach stood out from conventional multistep approaches and provided an efficient route towards several original derivatives that were structural analogs of naturally occurring balsacones, uvaretin, and erioschalcones. This study disclosed the influence of the *C*-alkylation pattern of the DHC **1** towards antimicrobial activity against *S. aureus*. Furthermore, it suggested that the substitution of the DHC had a critical impact on the anti-Gram-negative properties of *C*-prenylated DHCs and on the cytotoxicity of *C*-benzylated DHCs. The strategy reported here gave a better insight towards the bioactivity of *C*-alkylated DHCs and its extension to other naturally occurring precursors could pave the way to the discovery of novel analogs with improved antibacterial and cytotoxic properties. 

## Figures and Tables

**Table 1:** Catalytic Cinnamylation of Dihydrochalcone **1**.

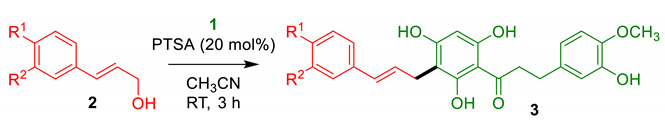		
Entry	Alcohol 2 (R ^1^, R ^2^)	Product 3 (Yield) ^1^
1	**2a** (OH, H)	**3a** (69%)
2	**2b** (OCH_3_, H)	**3b** (49%)
3	**2c** (OCH_3_, OCH_3_)	**3c** (61%)
4 ^2^	**2d** (H, H)	**3d** (72%)
5 ^2^	**2e** (Cl, H)	**3e** (30%)

^1^ Isolated yield. ^2^ The reaction was carried out in 2 h at reflux.

**Table 2:** Catalytic benzylation of dihydrochalcone **1**.

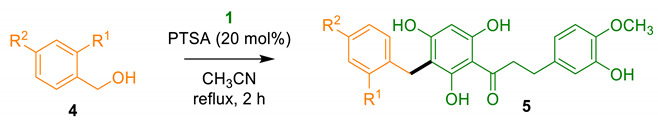		
Entry	Alcohol 4 (R ^1^, R ^2^)	Product 5 (Yield) ^1^
1	**4a** (OH, H)	**5a** (61%)
2	**4b** (H, OH)	**5b** (74%)
3 ^2^	**4c** (H, OCH_3_)	**5c** (36%)
4	**4d** (H, Cl)	**5d** (36%)

^1^ Isolated yield. ^2^ The reaction was carried out in 3 h at room temperature.

**Table 3:** Catalytic Prenylation of Dihydrochalcone **1**.

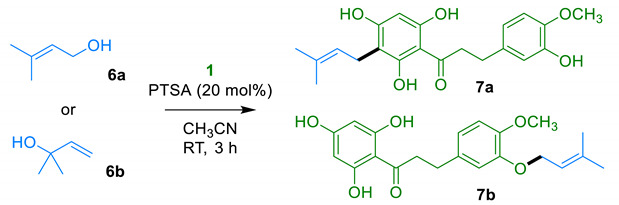		
Entry	Alcohol 6 (Equiv)	Product Ratio 7a:7b ^1^ (Yield) ^2^
1	**6a** (0.25 equiv)	1:1 (40%)
2	**6a** (0.50 equiv)	1.3:1 (48%)
3	**6a** (1.0 equiv)	2.1:1 (46%)
4	**6b** (0.25 equiv)	1.4:1 (58%)
5	**6b** (0.50 equiv)	1.4:1 (44%)
6	**6b** (1.0 equiv)	1.9:1 (26%)

^1^ The **7a**:**7b** ratio was determined on the ^1^H NMR spectrum of the isolated mixture. ^2^ Isolated yield of the mixture of **7a** and **7b**.

**Table 4:** Antibacterial and Cytotoxic Activity of Compounds **3a**–**e**, **5a**–**d**, **7a**–**b**, and **8a**.

Entry	Product	MIC_90_ (µM) ^1^		IC_50_ (µM) ^1,6^		
		*E. coli*	*S. aureus*	A-549	DLD-1	WS-1
1	**3a**	>100	11.6 ± 0.2	36 ± 3	28 ± 5	57 ± 4
2	**3b**	>100	5.7 ± 0.1	47 ± 6	26 ± 8	56 ± 5
3	**3c**	>100	6.0 ± 0.1	30 ± 3	36 ± 4	57 ± 6
4	**3d**	>100	5 ± 0.9	43 ± 4	35 ± 6	58 ± 3
5	**3e**	>100	2.6 ± 0.4	44 ± 3	44 ± 7	59 ± 5
6	**5a**	>100	12 ± 1	>100	>100	>100
7	**5b**	>100	20 ± 1	52 ± 9	64 ± 5	60 ± 5
8	**5c**	>100	4.2 ± 0.5	25 ± 2	29 ± 3	39 ± 4
9	**5d**	>100	2.3 ± 0.1	38 ± 2	46 ± 3	44 ± 4
10	**7a**	>100	80 ± 3	>100	>100	>100
11	**7b**	>100	98 ± 1	>100	>100	>100
12	**8a**	>100	2.5 ± 0.3	28 ± 2	21 ± 3	35 ± 4
13	Balsacone A ^2^	>100	3.3 ± 0.2	44 ± 4	31 ± 5	56 ± 4
14	Gentamicin ^2^	0.083 ± 0.007 ^3^	0.12 ± 0.01 ^3^	n. d. ^4^	n. d.	n. d.
15	Etoposide ^5^	n. d.	n. d.	0.56 ± 0.04	0.96 ± 0.08	10 ± 5

^1^ MIC_90_ and IC_50_ values ± SD (*n* = 3) are representative of two different experiments. ^2^ Used as a positive control for antibacterial assays. ^3^ Expressed in µg/mL. ^4^ N. d.: not determined. ^5^ Used as a positive control for cytotoxic assays. ^6^ Values > 20 µM were considered as non-toxic.

## Data Availability

The data presented in this study are available in the Materials and methods section and in the Supplementary Material.

## Supplementary Materials

The following are available online at https://www.mdpi.com/article/10.3390/antibiotics10060620/s1, Figures S1–S12: NMR spectra of compounds **3a–e’**; Figures S13–S20: NMR spectra of compounds **5a–d**; Figures S21–S24: NMR spectra of compounds **7a–b**; Figures S25–S28: NMR spectra of compounds **8a–b**.

## References

[1] Ibdah M., Martens S., Gang D.R. (2018). Biosynthetic pathway and metabolic engineering of plant dihydrochalcones. J. Agric. Food Chem..

[2] Stompor M., Broda D., Bajek-Bil A. (2019). Dihydrochalcones: Methods of acquisition and pharmacological properties—A first systematic review. Molecules.

[3] Veitch N.C., Grayer R.J. (2011). Flavonoids and their glycosides, including anthocyanins. Nat. Prod. Rep..

[4] Snijman P.W., Joubert E., Ferreira D., Li X.-C., Ding Y., Green I.R., Gelderblom W.C.A. (2009). Antioxidant activity of the dihydrochalcones aspalathin and nothofagin and their corresponding flavones in relation to other rooibos (*Aspalathus linearis*) flavonoids, epigallocatechin gallate, and Trolox. J. Agric. Food Chem..

[5] Lavoie S., Legault J., Simard F., Chiasson É., Pichette A. (2013). New antibacterial dihydrochalcone derivatives from buds of *Populus balsamifera*. Tetrahedron Lett..

[6] Simard F., Legault J., Lavoie S., Pichette A. (2014). Balsacones D-I, dihydrocinnamoyl flavans from *Populus balsamifera* buds. Phytochemistry.

[7] Simard F., Gauthier C., Chiasson É., Lavoie S., Mshvildadze V., Legault J., Pichette A. (2015). Antibacterial balsacones J–M, hydroxycinnamoylated dihydrochalcones from *Populus balsamifera* buds. J. Nat. Prod..

[8] Simard F., Gauthier C., Legault J., Lavoie S., Mshvildadze V., Pichette A. (2016). Structure elucidation of anti-methicillin resistant *Staphylococcus aureus* (MRSA) flavonoids from balsam poplar buds. Bioorg. Med. Chem..

[9] Muhammad I., Waterman P.G. (1985). Chemistry of the Annonaceae, part 18. Benzylated indoles and dihydrochalcones in *Uvaria angolensis* from Tanzania. J. Nat. Prod..

[10] Nkunya M.H.H., Weenen H., Renner C., Waibel R., Achenbach H. (1993). Benzylated dihydrochalcones from *Uvaria leptocladon*. Phytochemistry.

[11] Prawat U., Chairerk O., Phupornprasert U., Salae A.-W., Tuntiwachwuttikul P. (2013). Two New *C*-benzylated Dihydrochalcone Derivatives from the Leaves of *Melodorum siamensis*. Planta Med..

[12] Awouafack M.D., Kouam S.F., Hussain H., Ngamga D., Tane P., Schulz B., Green I.R., Krohn K. (2008). Antimicrobial Prenylated Dihydrochalcones from *Eriosema glomerata*. Planta Med..

[13] Rivière C., Rahman A. (2016). Chapter 7—Dihydrochalcones: Occurrence in the Plant Kingdom, Chemistry and Biological Activities. Studies in Natural Products Chemistry.

[14] Kozłowska J., Potaniec B., Żarowska B., Anioł M. (2018). Microbial transformations of 4′-methylchalcones as an efficient method of obtaining novel alcohol and dihydrochalcone derivatives with antimicrobial activity. RSC Adv..

[15] Gosch C., Halbwirth H., Stich K. (2010). Phloridzin: Biosynthesis, distribution and physiological relevance in plants. Phytochemistry.

[16] Horowitz R.M., Gentili B. (1969). Taste and structure in phenolic glycosides. J. Agric. Food Chem..

[17] Alsarraf J., Bilodeau J.-F., Legault J., Simard F., Pichette A. (2020). Exploring the Biomass-Derived Chemical Space Emerging from Natural Dihydrochalcones through the Single-Step Hemisynthesis of Antibacterial Balsacones. ACS Sustain. Chem. Eng..

[18] Bélanger A., Grenier A., Simard F., Gendreau I., Pichette A., Legault J., Pouliot R. (2020). Dihydrochalcone Derivatives from *Populus balsamifera* L. Buds for the Treatment of Psoriasis. Int. J. Mol. Sci..

[19] Côté H., Pichette A., Simard F., Ouellette M.-E., Ripoll L., Mihoub M., Grimard D., Legault J. (2019). Balsacone C, a New Antibiotic Targeting Bacterial Cell Membranes, Inhibits Clinical Isolates of Methicillin-Resistant *Staphylococcus aureus* (MRSA) Without Inducing Resistance. Front. Microbiol..

[20] Sanz R., Martínez A., Miguel D., Álvarez-Gutiérrez J.M., Rodríguez F. (2006). Brønsted Acid-Catalyzed Nucleophilic Substitution of Alcohols. Adv. Synth. Catal..

[21] Bandini M., Tragni M. (2009). π-Activated alcohols: An emerging class of alkylating agents for catalytic Friedel–Crafts reactions. Org. Biomol. Chem..

[22] Kumar R., Van der Eycken E.V. (2013). Recent approaches for C–C bond formation via direct dehydrative coupling strategies. Chem. Soc. Rev..

[23] Rodrigues T., Reker D., Schneider P., Schneider G. (2016). Counting on natural products for drug design. Nat. Chem..

[24] Hufford C.D., Oguntimein B.O. (1980). Dihydrochalcones from *Uvaria angolensis*. Phytochemistry.

[25] Hufford C.D., Oguntimein B.O. (1982). New Dihydrochalcones and Flavanones From *Uvaria Angolensis*. J. Nat. Prod..

[26] Hufford C.D., Oguntimein B.O., Shoolery J.N. (1987). Angoluvarin, an antimicrobial dihydrochalcone from *Uvaria angolensis*. J. Org. Chem..

[27] Dallman J., Lansakara A., Nguyen T., Weeramange C., Hulangamuwa W., Rafferty R.J. (2019). The winding road of the uvaretin class of natural products: From total synthesis to bioactive agent discovery. MedChemComm.

[28] Terao J., Mukai R. (2014). Prenylation modulates the bioavailability and bioaccumulation of dietary flavonoids. Arch. Biochem. Biophys..

[29] Botta B., Vitali A., Menendez P., Misiti D., Delle Monache G. (2005). Prenylated flavonoids: Pharmacology and biotechnology. Curr. Med. Chem..

[30] Quideau S., Ralph J. (1992). Facile large-scale synthesis of coniferyl, sinapyl, and *p*-coumaryl alcohol. J. Agric. Food Chem..

[31] Malterud K.E., Undheim J., Erdal J.E. (1985). Synthesis of uvaretin, an antitumour and antimicrobial flavonoid. Tetrahedron Lett..

[32] Sanz R., Martínez A., Guilarte V., Álvarez-Gutiérrez J.M., Rodríguez F. (2007). The Ritter Reaction under Truly Catalytic Brønsted Acid Conditions. Eur. J. Org. Chem..

[33] Yu X., Li S.-M. (2011). Prenylation of Flavonoids by Using a Dimethylallyltryptophan Synthase, 7-DMATS, from *Aspergillus fumigatus*. ChemBioChem.

[34] Qi J., Porco J.A. (2007). Rapid Access to Polyprenylated Phloroglucinols via Alkylative Dearomatization−Annulation:  Total Synthesis of (±)-Clusianone. J. Am. Chem. Soc..

[35] Yu Q., Ravu R.R., Xu Q.-M., Ganji S., Jacob M.R., Khan S.I., Yu B.-Y., Li X.-C. (2015). Antibacterial Prenylated Acylphloroglucinols from *Psorothamnus fremontii*. J. Nat. Prod..

[36] Khupse R.S., Erhardt P.W. (2007). Total Synthesis of Xanthohumol. J. Nat. Prod..

[37] Tran T.D., Olsson M.A., McMillan D.J., Cullen J.K., Parsons P.G., Reddell P.W., Ogbourne S.M. (2020). Potent antibacterial prenylated acetophenones from the Australian endemic plant *Acronychia crassipetala*. Antibiotics.

[38] Fulmer G.R., Miller A.J.M., Sherden N.H., Gottlieb H.E., Nudelman A., Stoltz B.M., Bercaw J.E., Goldberg K.I. (2010). NMR Chemical shifts of trace impurities: Common laboratory solvents, organics, and gases in deuterated solvents relevant to the organometallic chemist. Organometallics.

[39] Gupte A., Buolamwini J.K. (2009). Synthesis and biological evaluation of phloridzin analogs as human concentrative nucleoside transporter 3 (hCNT3) inhibitors. Bioorg. Med. Chem. Lett..

[40] Banfi E., Scialino G., Monti-Bragadin C. (2003). Development of a microdilution method to evaluate *Mycobacterium tuberculosis* drug susceptibility. J. Antimicrob. Chemother..

[41] Rage R., Mitchen J., Wilding G. (1990). DNA fluorometric assay in 96-well tissue culture plates using Hoechst 33258 after cell lysis by freezing in distilled water. Anal. Biochem..

