# Supplementary material for: Hemisynthesis and Biological Evaluation of Cinnamylated, Benzylated, and Prenylated Dihydrochalcones from a Common Bio-Sourced Precursor
Source: Antibiotics (Basel). 2021 May 22;10(6):620. doi: 10.3390/antibiotics10060620 (PMC8224620; doi:10.3390/antibiotics10060620)

antibiotics

Hemisynthesis and Biological Evaluation of Cinnamylated, Benzylated, and Prenylated Dihydrochalcones from a Common Bio-Sourced Precursor

Anne Ardaillou, Jérôme Alsarraf *, Jean Legault, François Simard and André Pichette *

Table of contents

1. ^{1}H and ^{13}C NMR Spectra of compound **3a** S1
2. ^{1}H and ^{13}C NMR Spectra of compound **3b** S3
3. ^{1}H and ^{13}C NMR Spectra of compound **3c** S5
4. ^{1}H and ^{13}C NMR Spectra of compound **3d** S7
5. ^{1}H and ^{13}C NMR Spectra of compound **3e** S9
6. ^{1}H and ^{13}C NMR Spectra of compound **3e’** S11
7. ^{1}H and ^{13}C NMR Spectra of compound **5a** S13
8. ^{1}H and ^{13}C NMR Spectra of compound **5b** S15
9. ^{1}H and ^{13}C NMR Spectra of compound **5c** S17
10. ^{1}H and ^{13}C NMR Spectra of compound **5d** S19
11. ^{1}H and ^{13}C NMR Spectra of compound **7a** S21
12. ^{1}H and ^{13}C NMR Spectra of compound **7b** S23
13. ^{1}H and ^{13}C NMR Spectra of compound **8a** S25
14. ^{1}H and ^{13}C NMR Spectra of compound **8b** S27

1. *^{1}H and ^{13}C NMR Spectra of compound **3a***

a. *Figure S1: ^{1}H NMR spectrum of compound **3a***

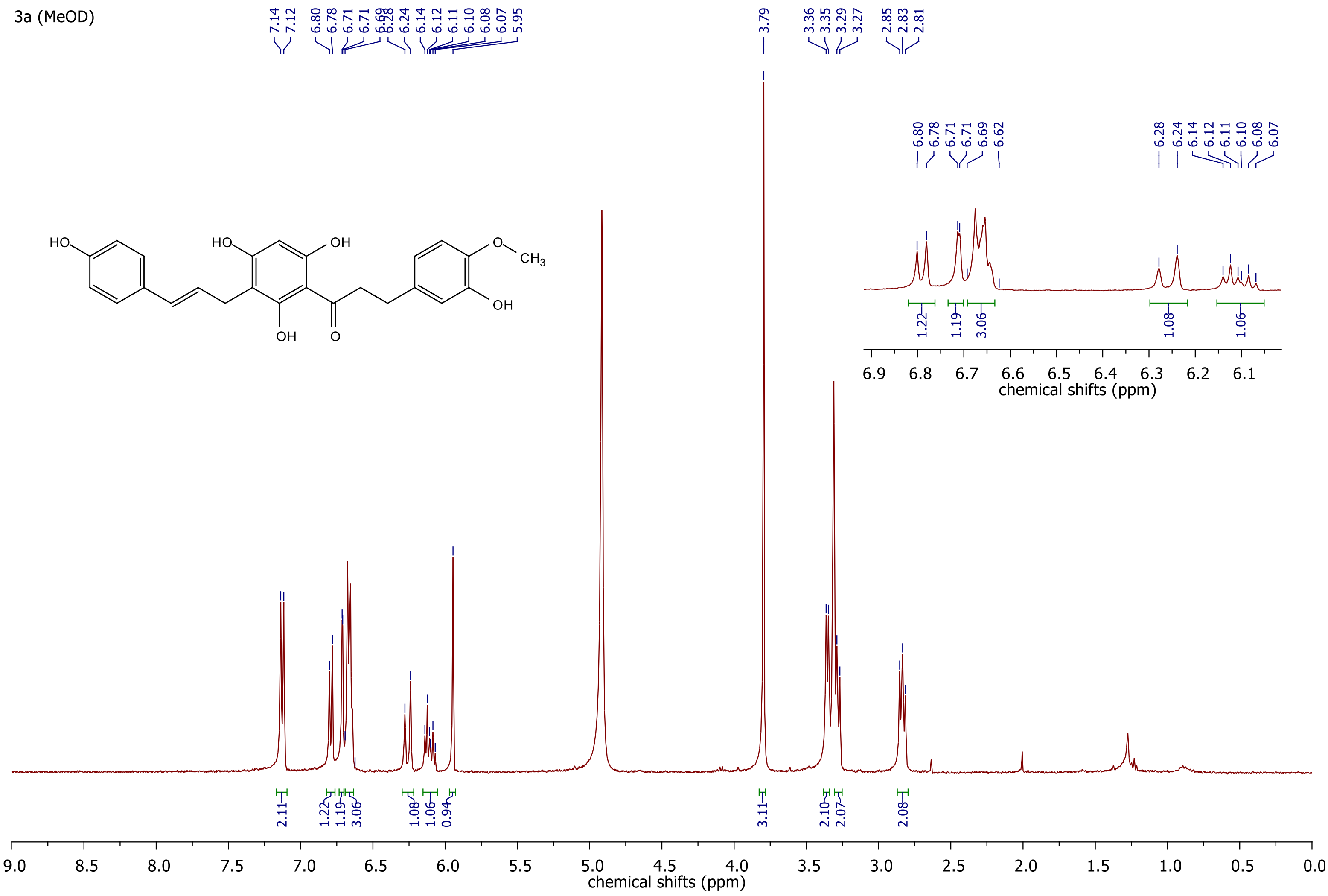

b. *Figure S2: ^{13}C NMR spectrum of compound **3a***

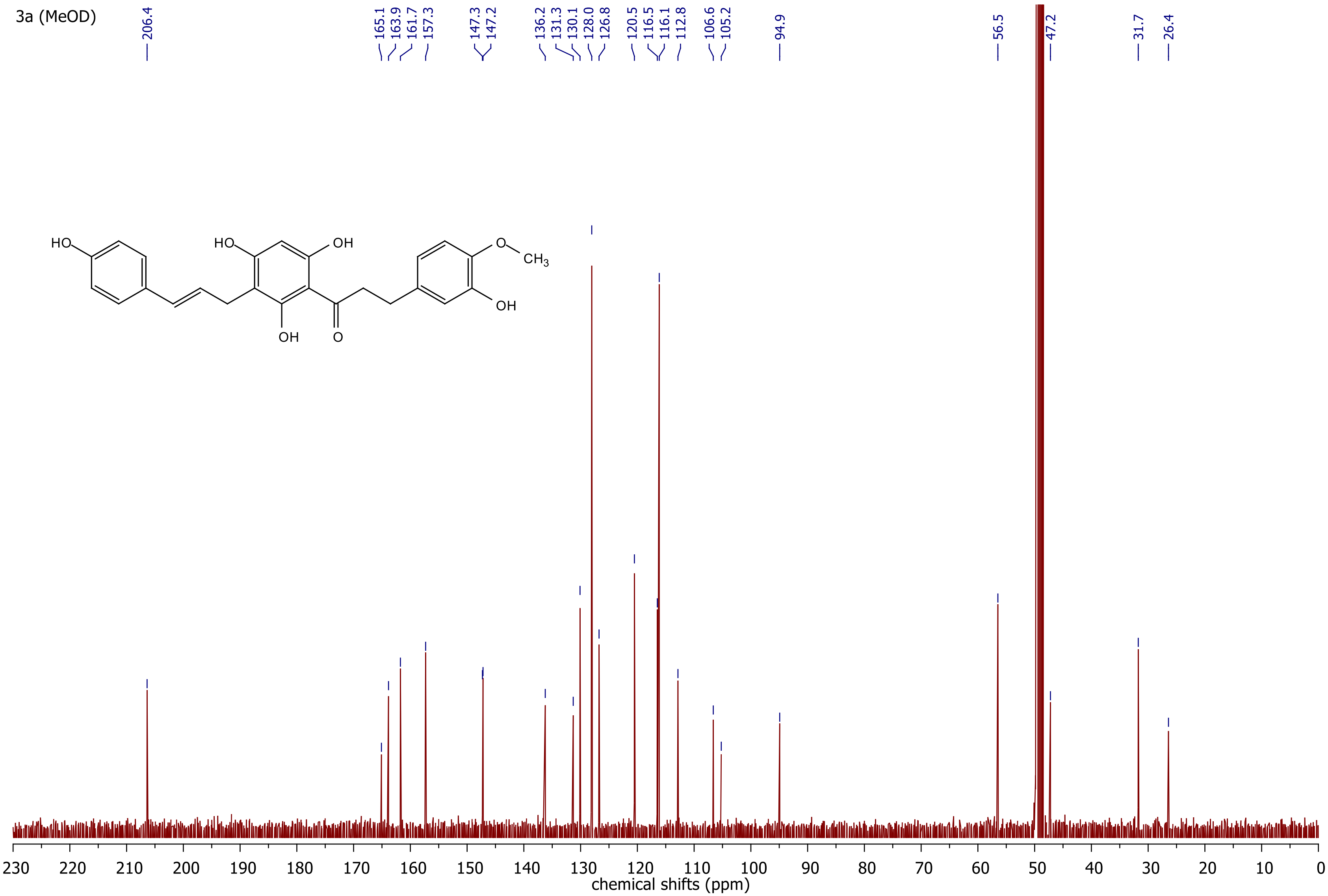

2. ^{1}H and ^{13}C NMR Spectra of compound ***3b***

a. *Figure S3: ^{1}H NMR spectrum of Compound **3b***

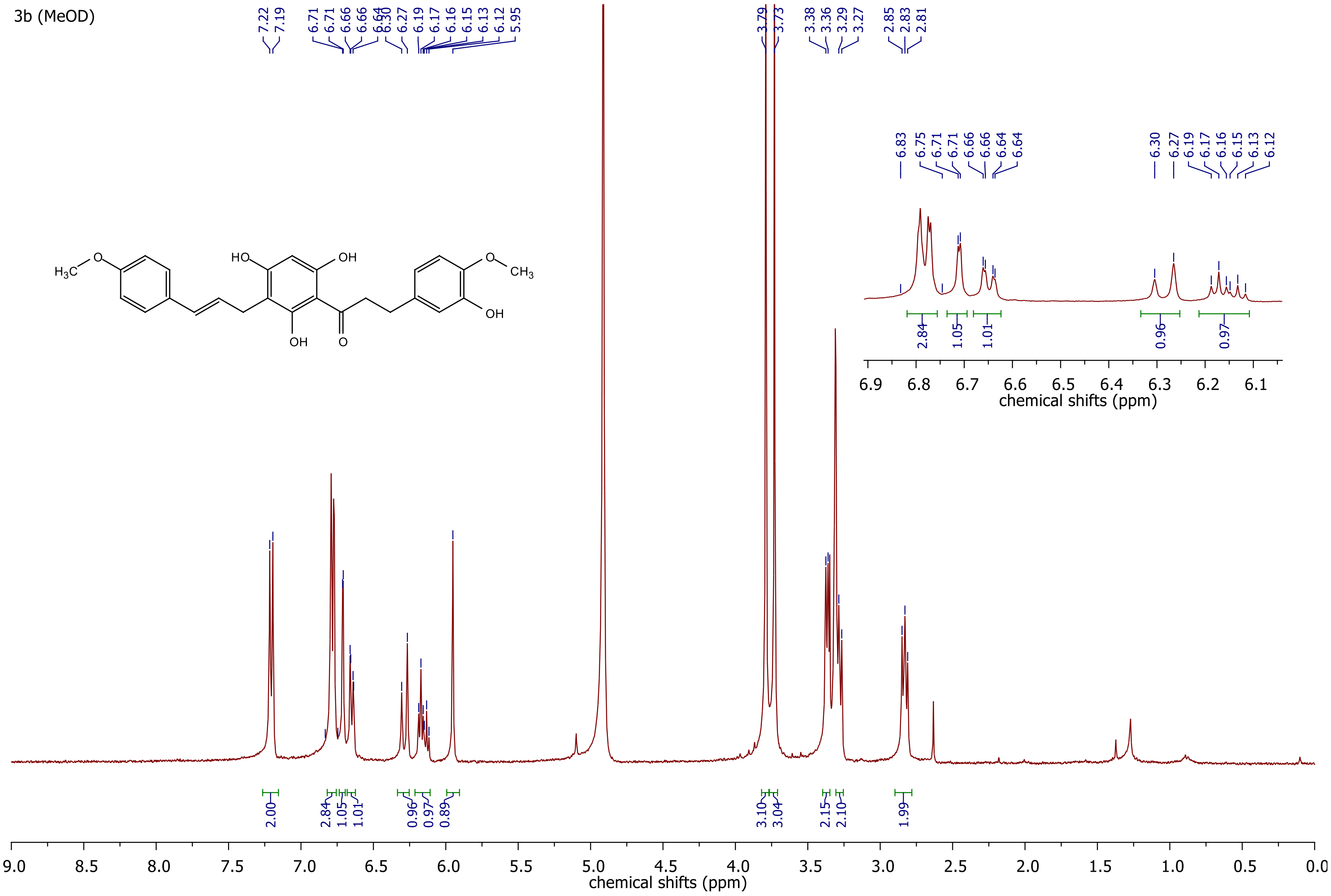

b. *Figure S4: ^{13}C NMR spectrum of Compound **3b***

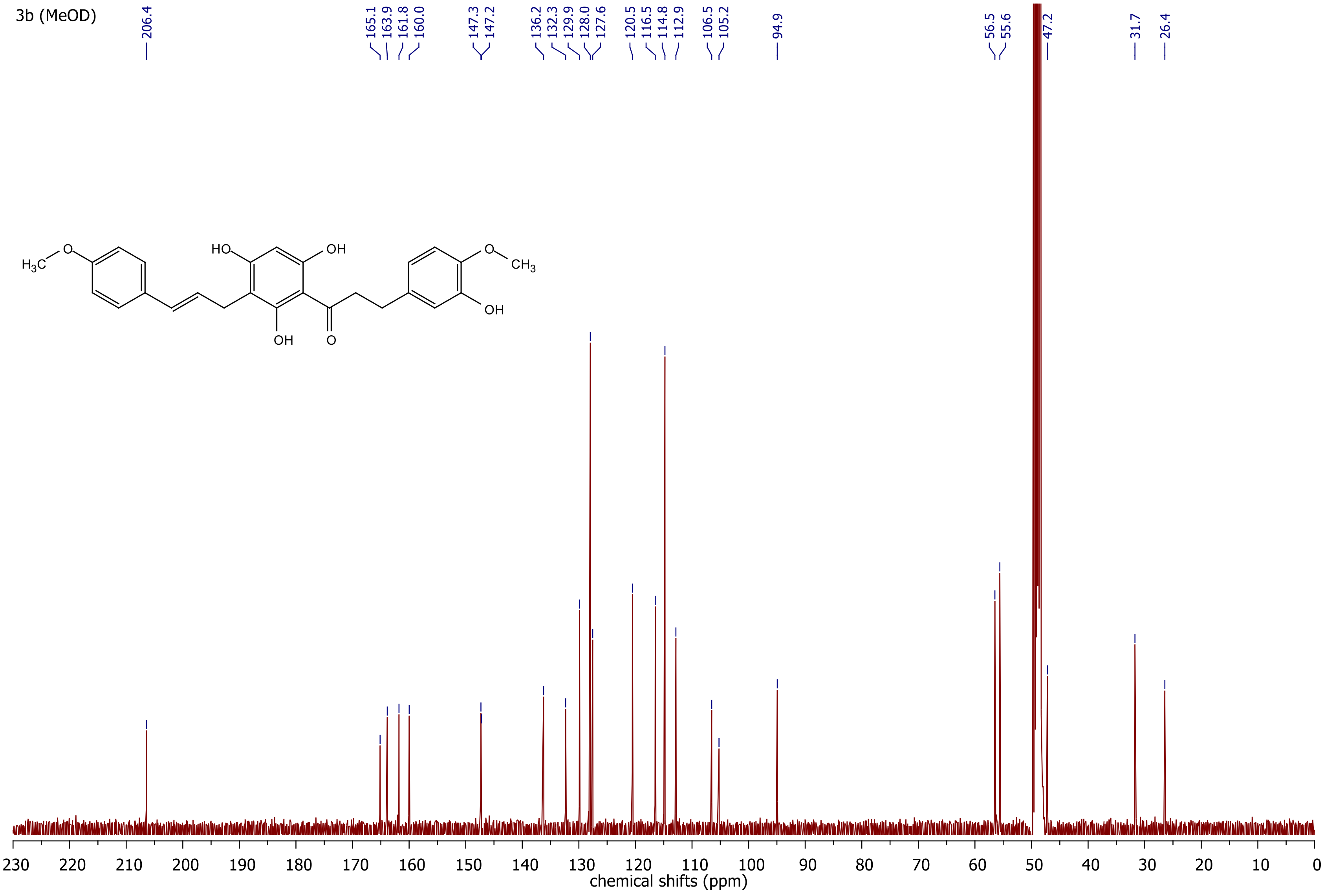

3. *^{1}H and ^{13}C NMR Spectra of compound* ***3c***

a. *Figure S5: ^{1}H NMR spectrum of Compound* ***3c***

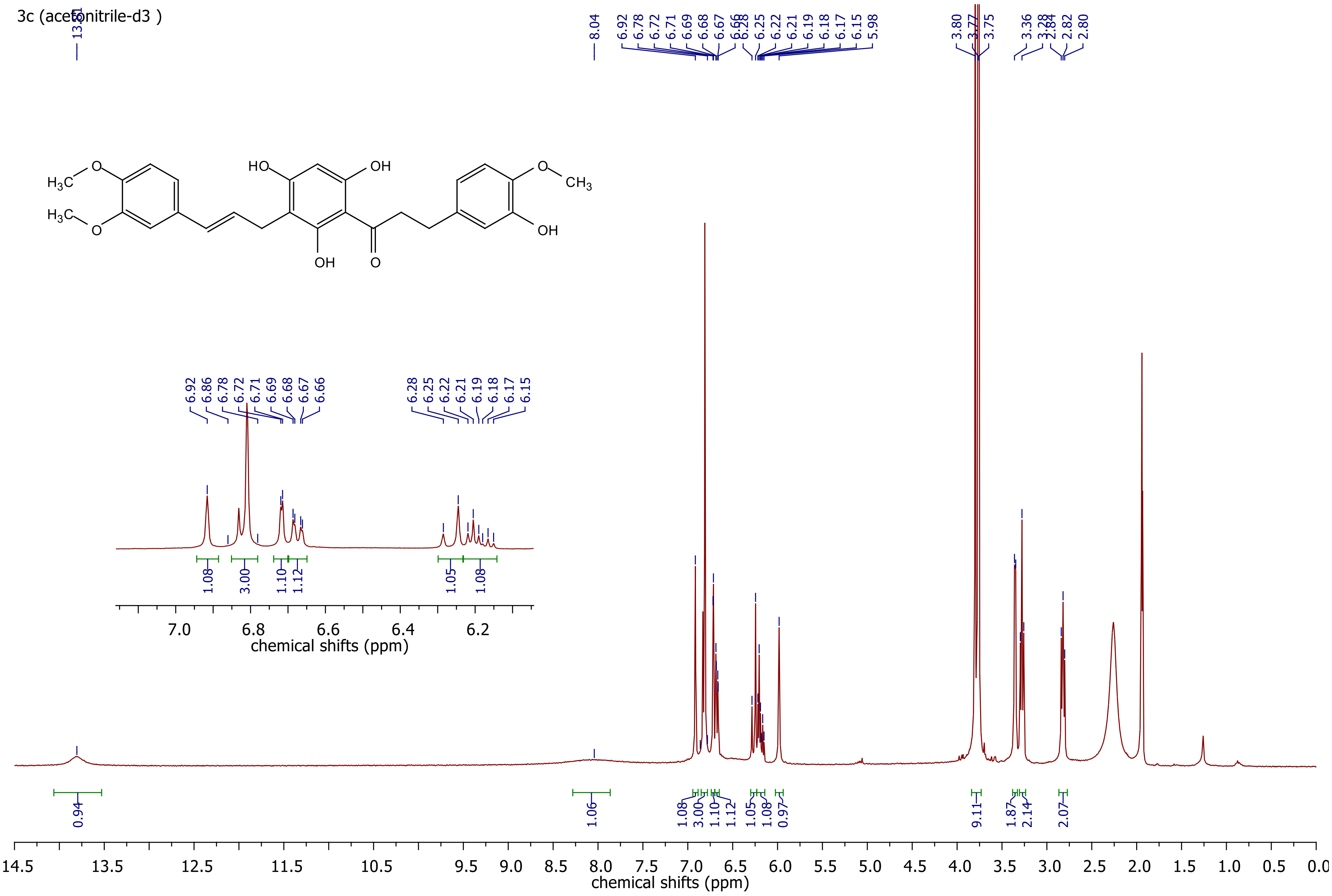

b. *Figure S6: ^{13}C NMR spectrum of Compound **3c***

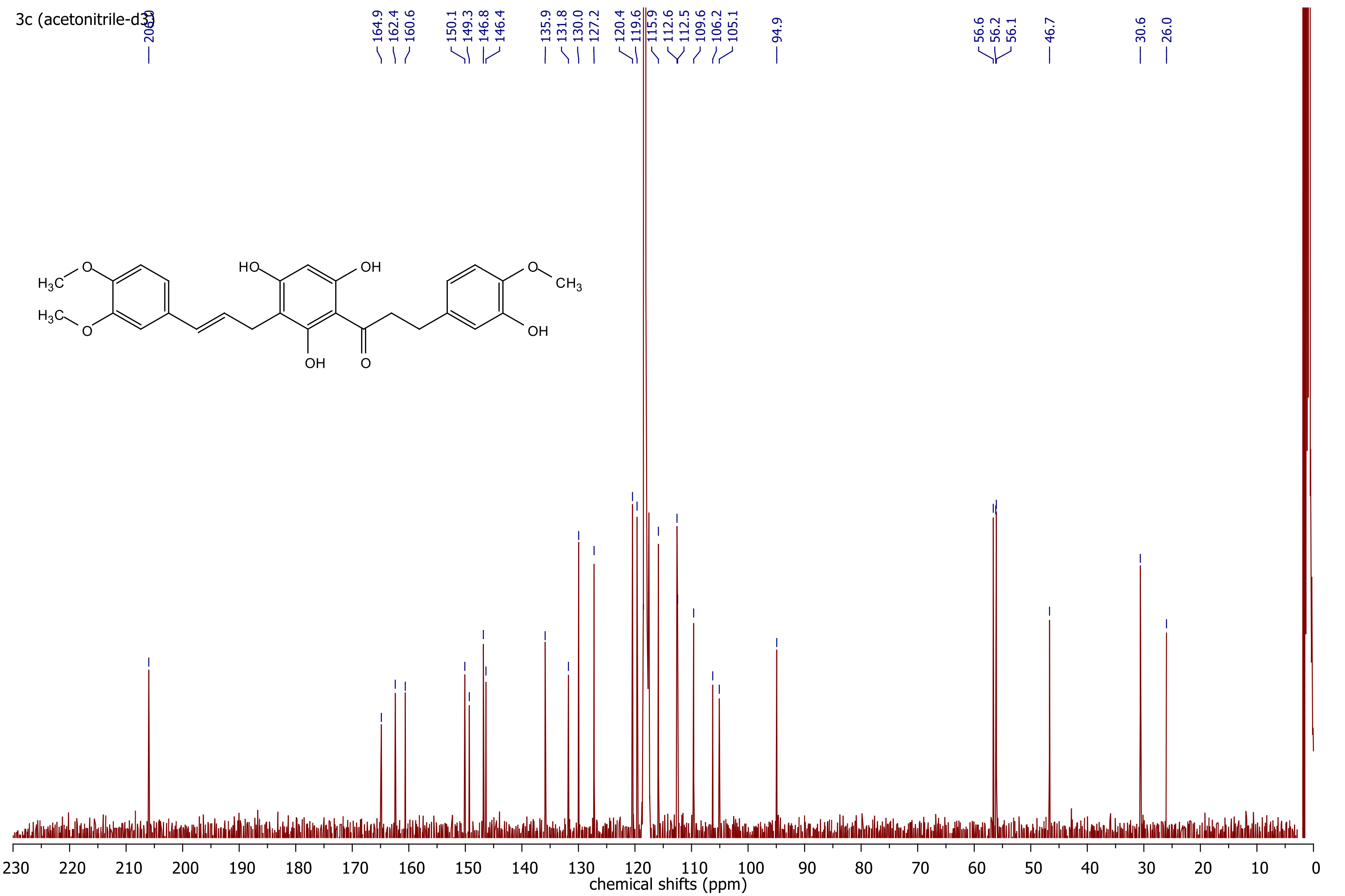

4. *^{1}H and ^{13}C NMR Spectra of compound* ***3d***

a. *Figure S7: ^{1}H NMR spectrum of Compound* ***3d***

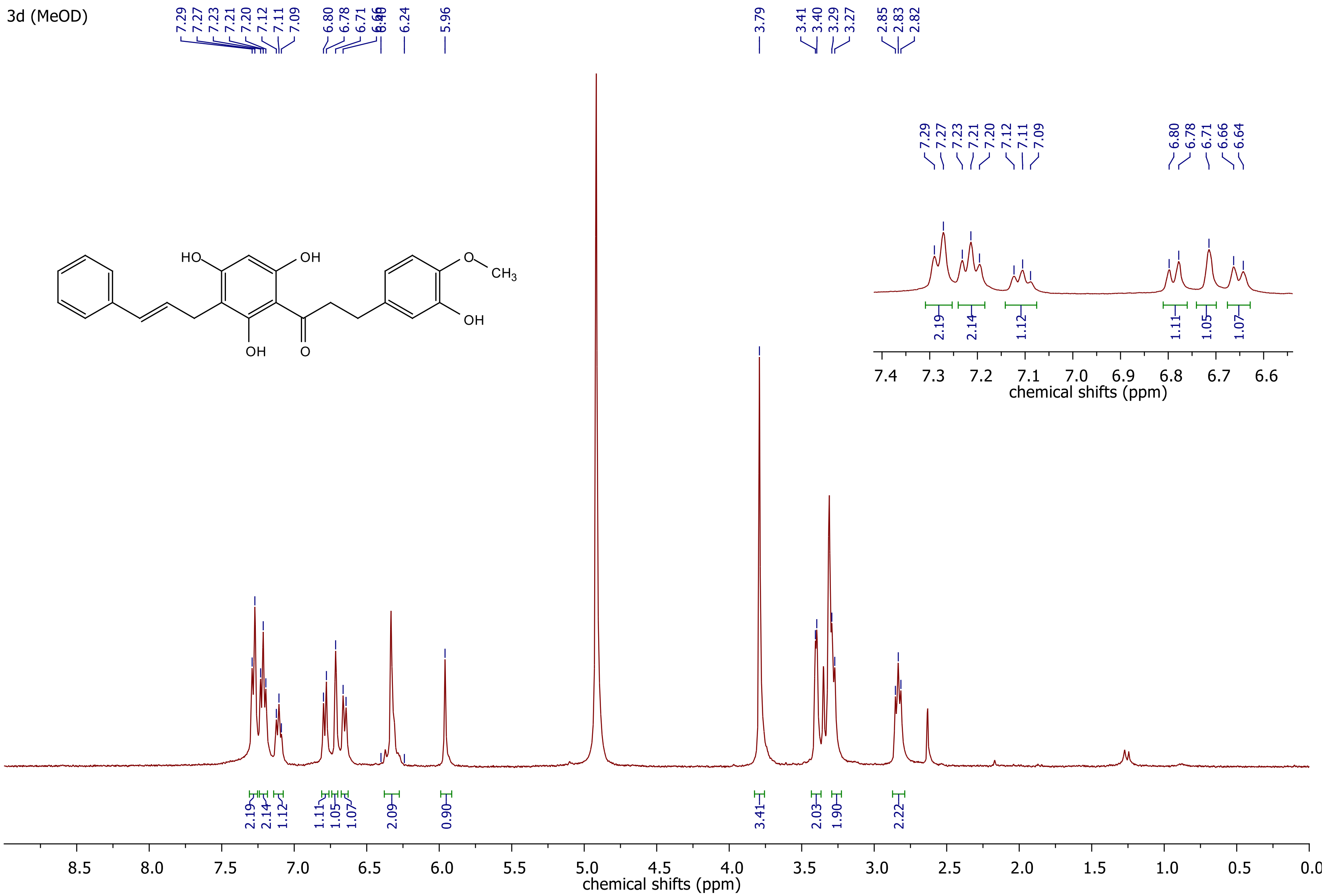

b. *Figure S8: ^{13}C NMR spectrum of Compound **3d***

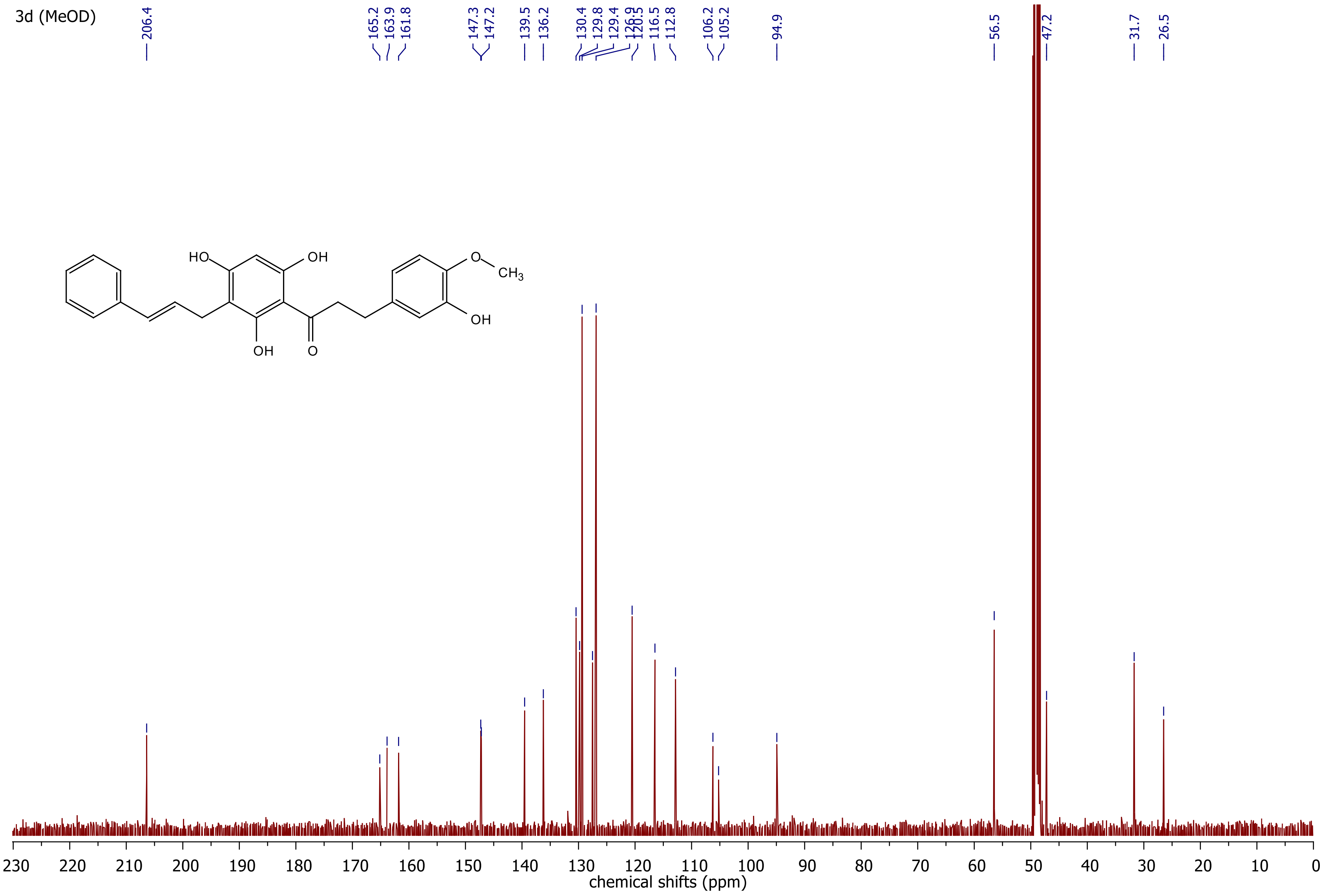

5. *^{1}H and ^{13}C NMR Spectra of compound **3e***

*a. Figure S9: ^{1}H NMR spectrum of Compound **3e***

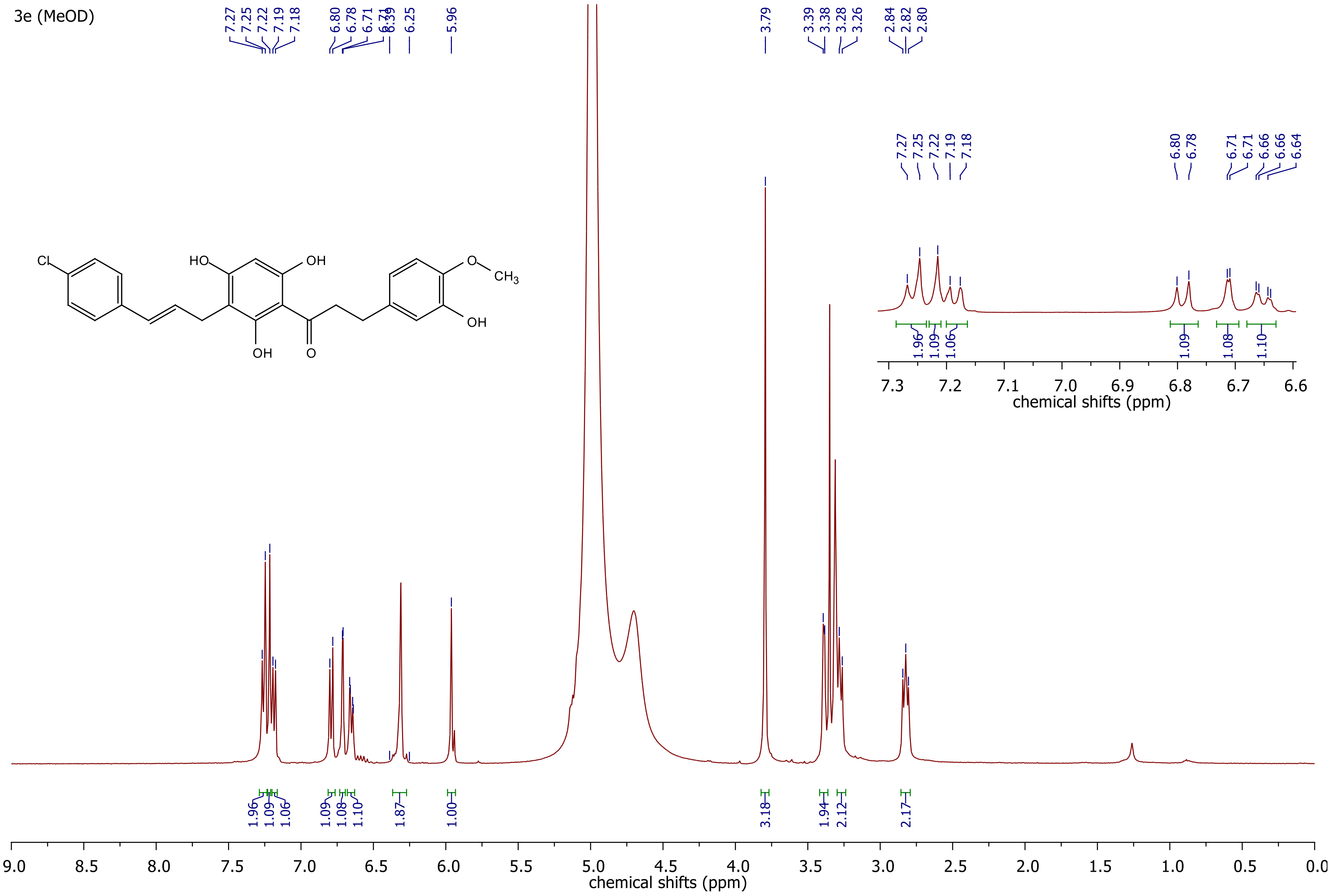

b. *Figure S10: ^{13}C NMR spectrum of Compound **3e***

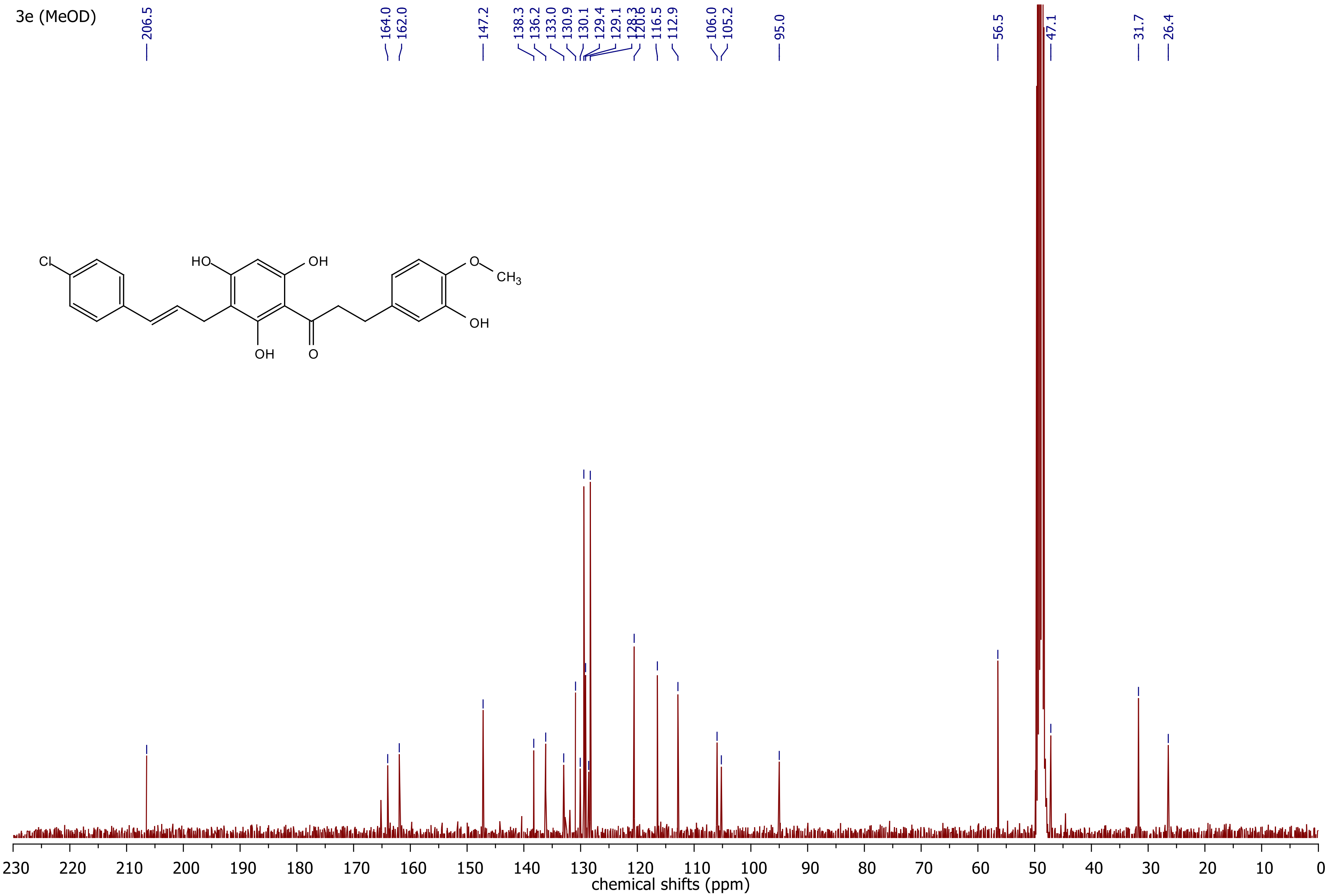

6. ^{1}H and ^{13}C NMR Spectra of compound **3e′**

a. Figure S11: ^{1}H NMR spectrum of Compound **3e′**

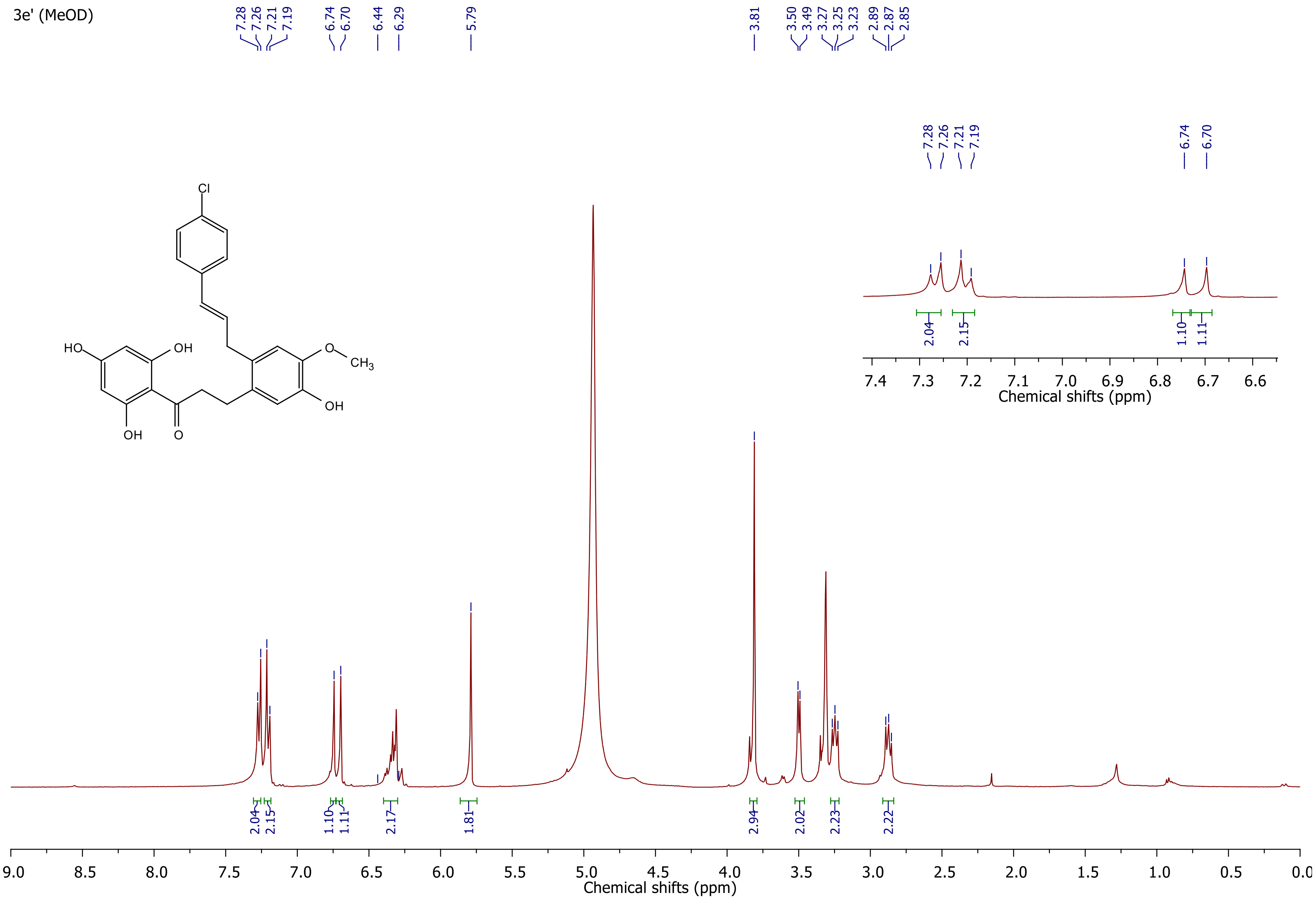

b. *Figure S12: ¹³C NMR spectrum of Compound **3e′***

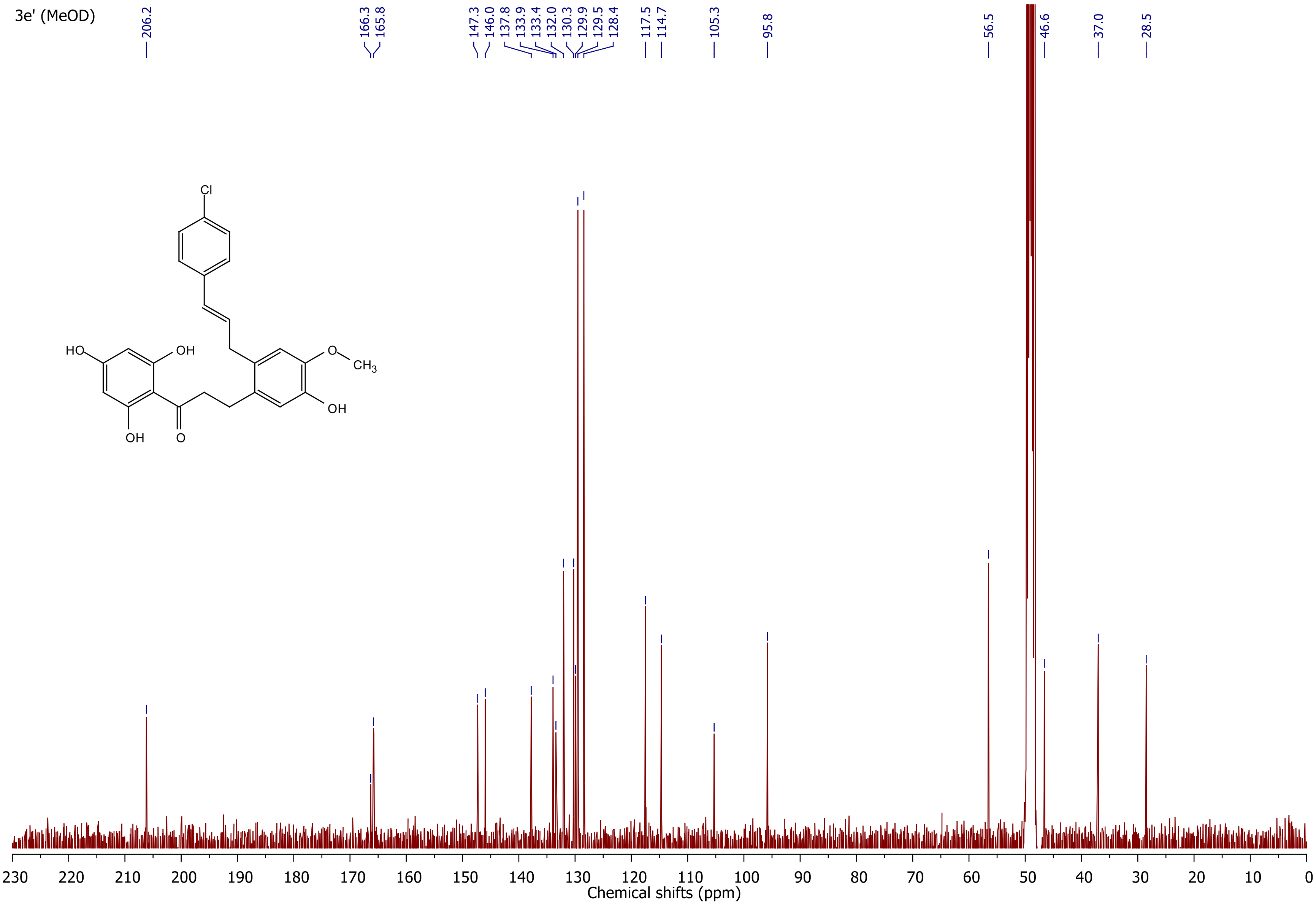

7. *^{1}H and ^{13}C NMR Spectra of compound **5a***

a. *Figure S13: ^{1}H NMR spectrum of Compound **5a***

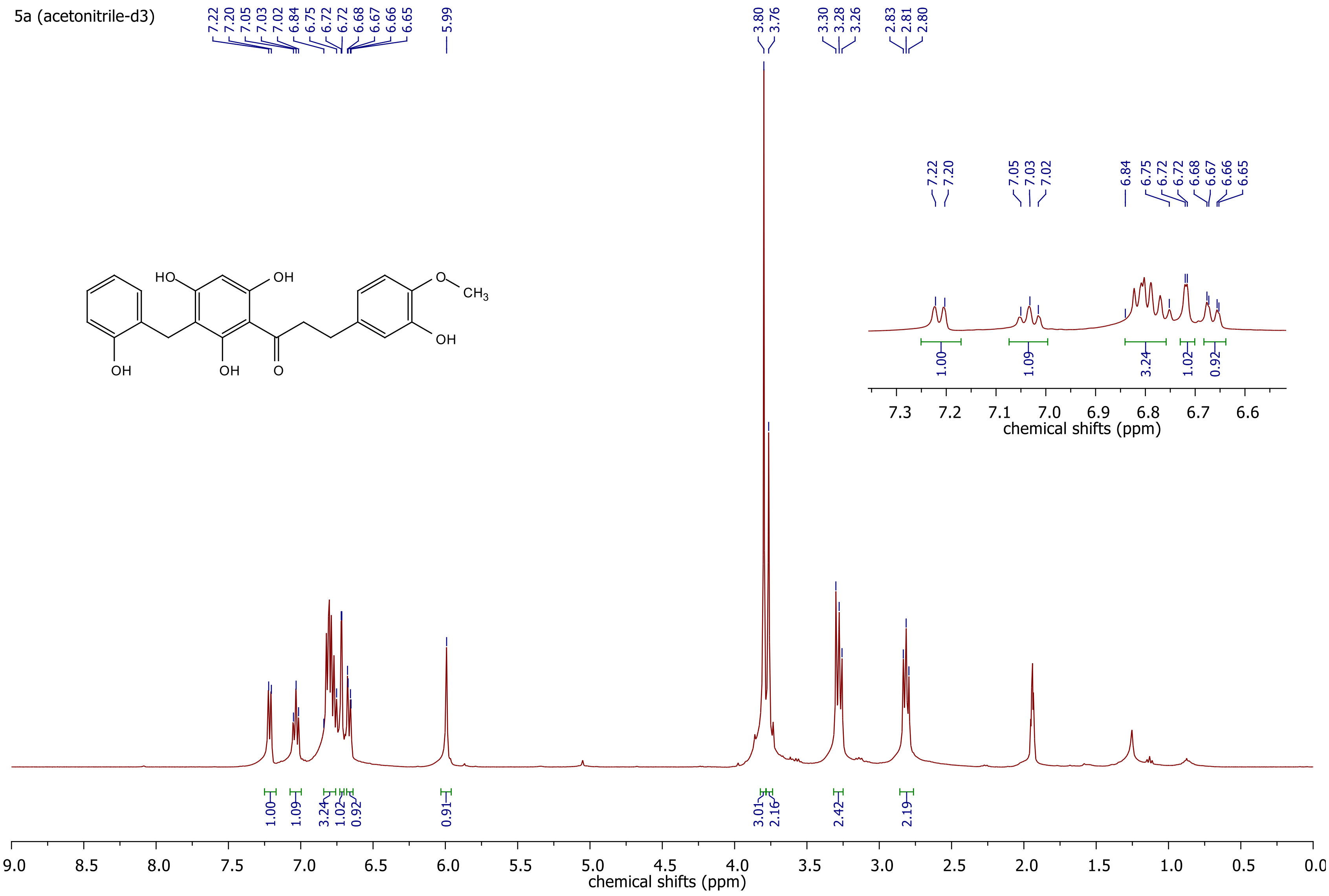

b. *Figure S14: ^{13}C NMR spectrum of Compound **5a***

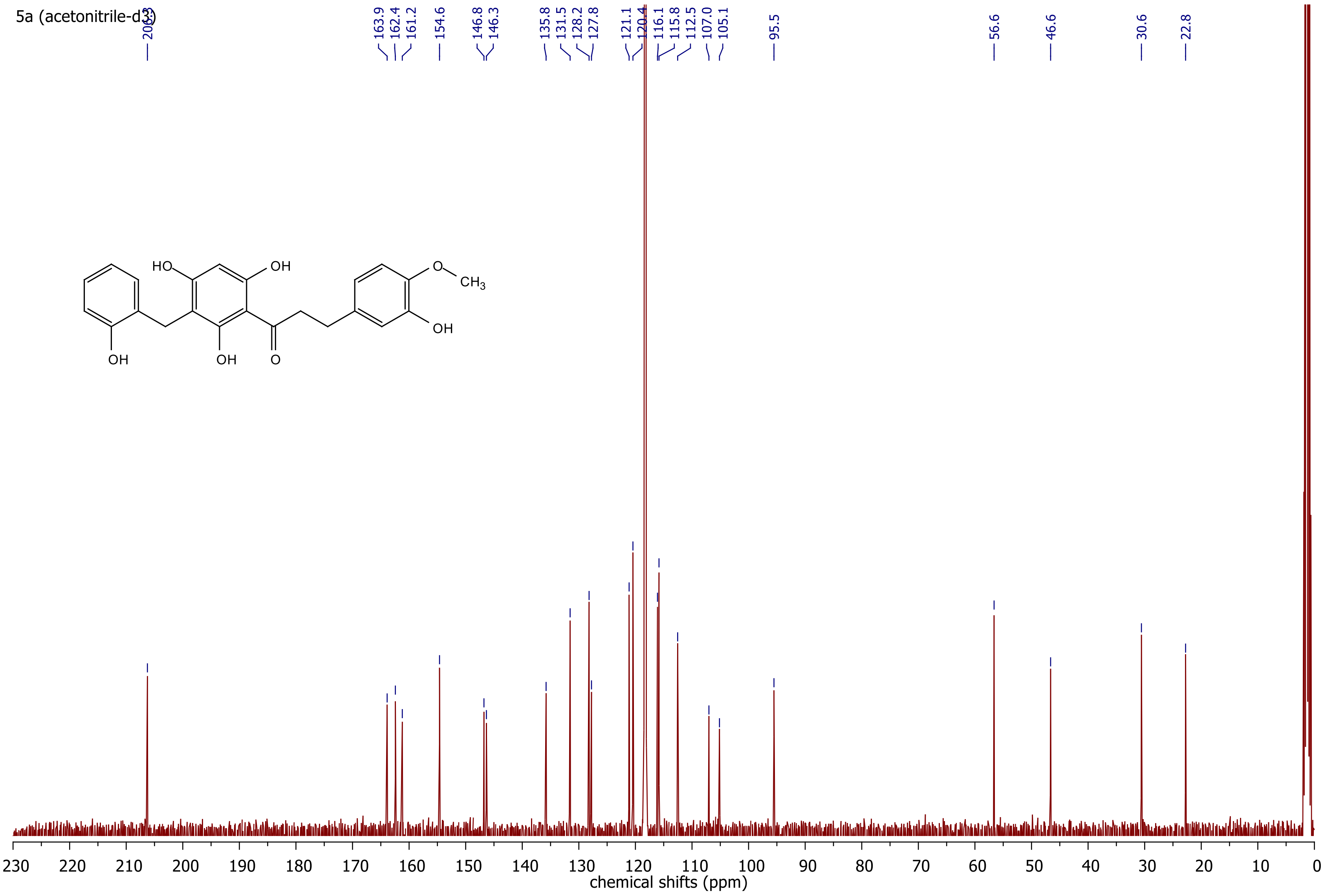

8. *^{1}H and ^{13}C NMR Spectra of compound **5b***

a. *Figure S15: ^{1}H NMR spectrum of Compound **5b***

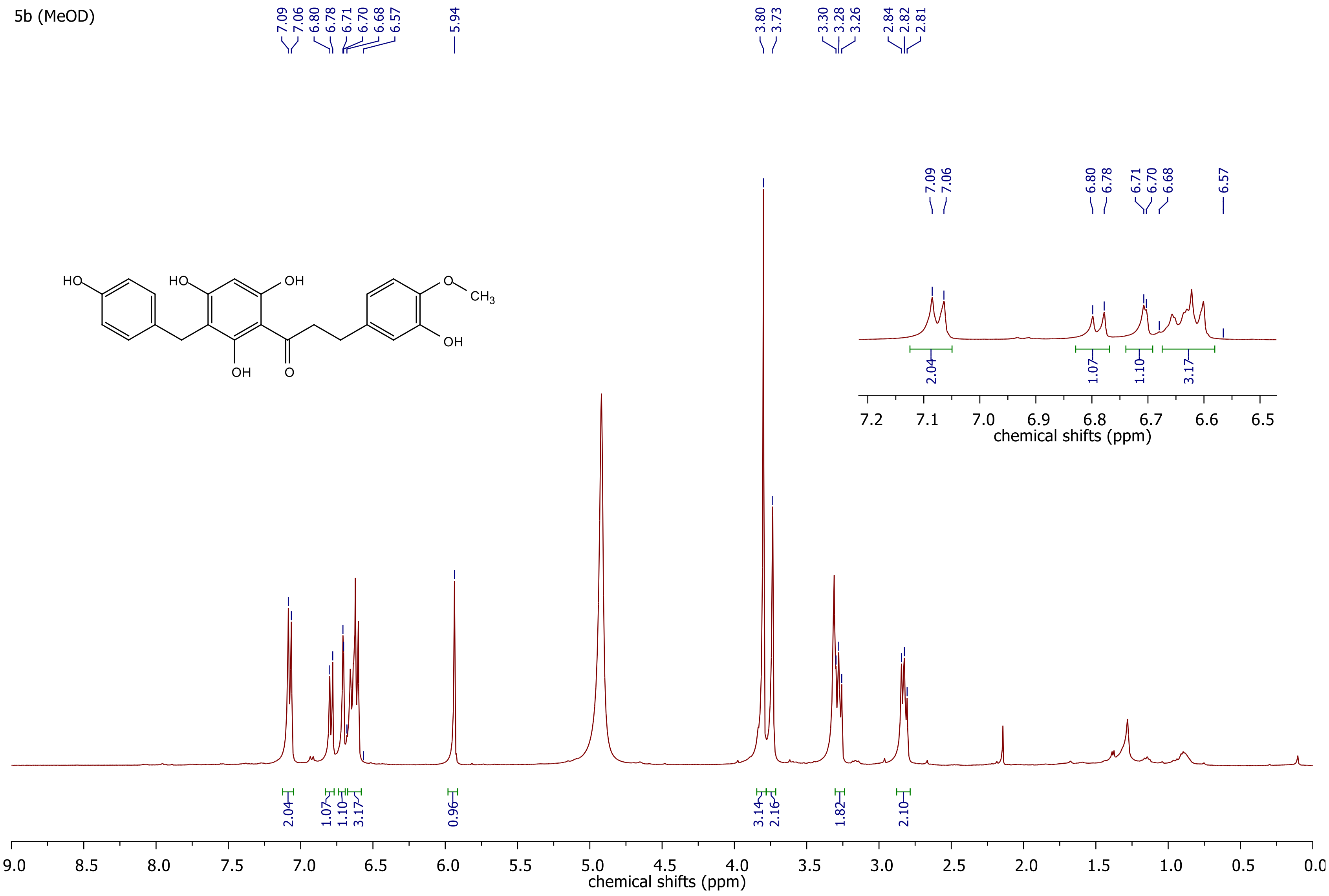

b. *Figure S16: ^{13}C NMR spectrum of Compound **5b***

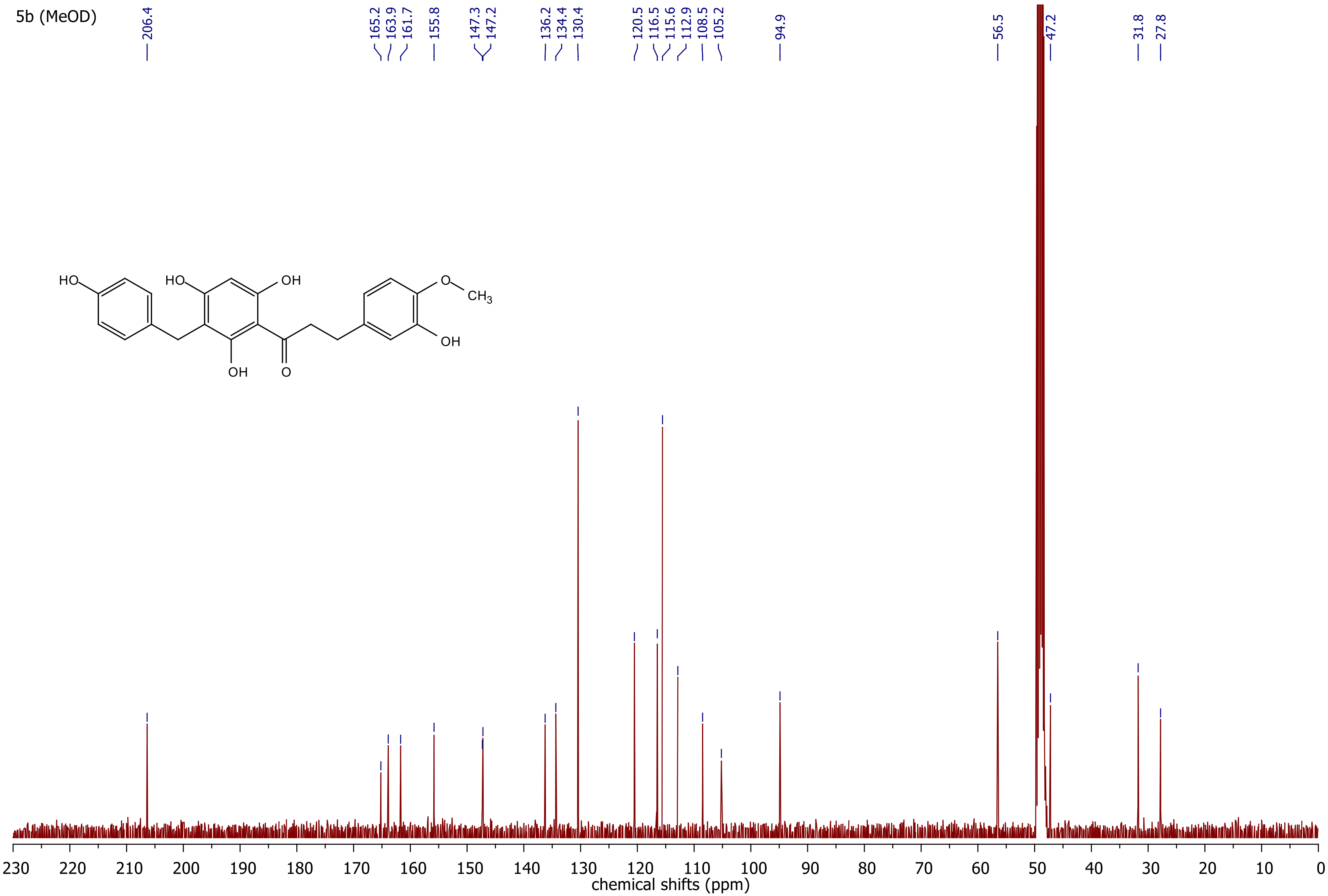

9. *^{1}H and ^{13}C NMR Spectra of compound **5c***

a. *Figure S17: ^{1}H NMR spectrum of Compound **5c***

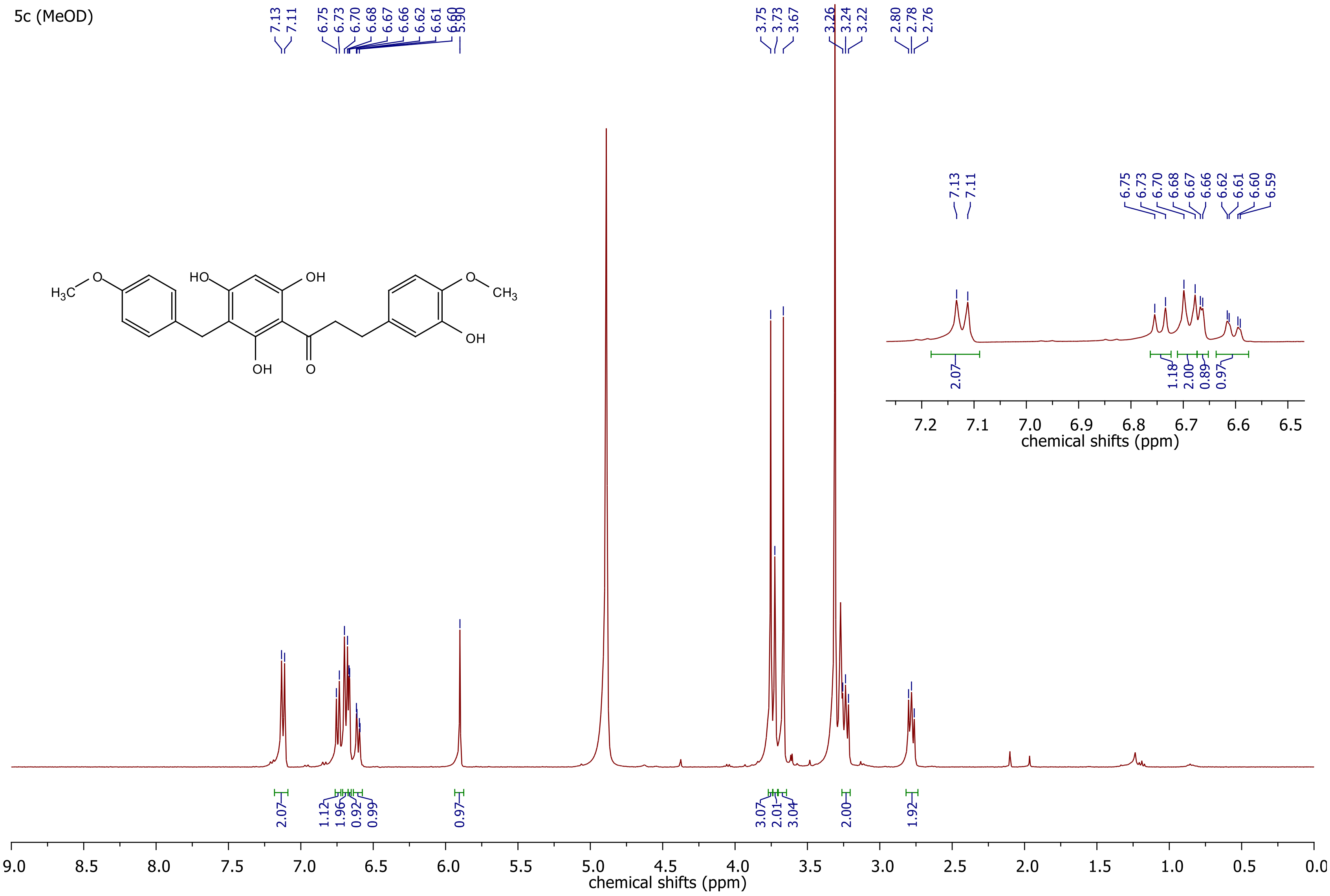

b. *Figure S18: ^{13}C NMR spectrum of Compound* ***5c***

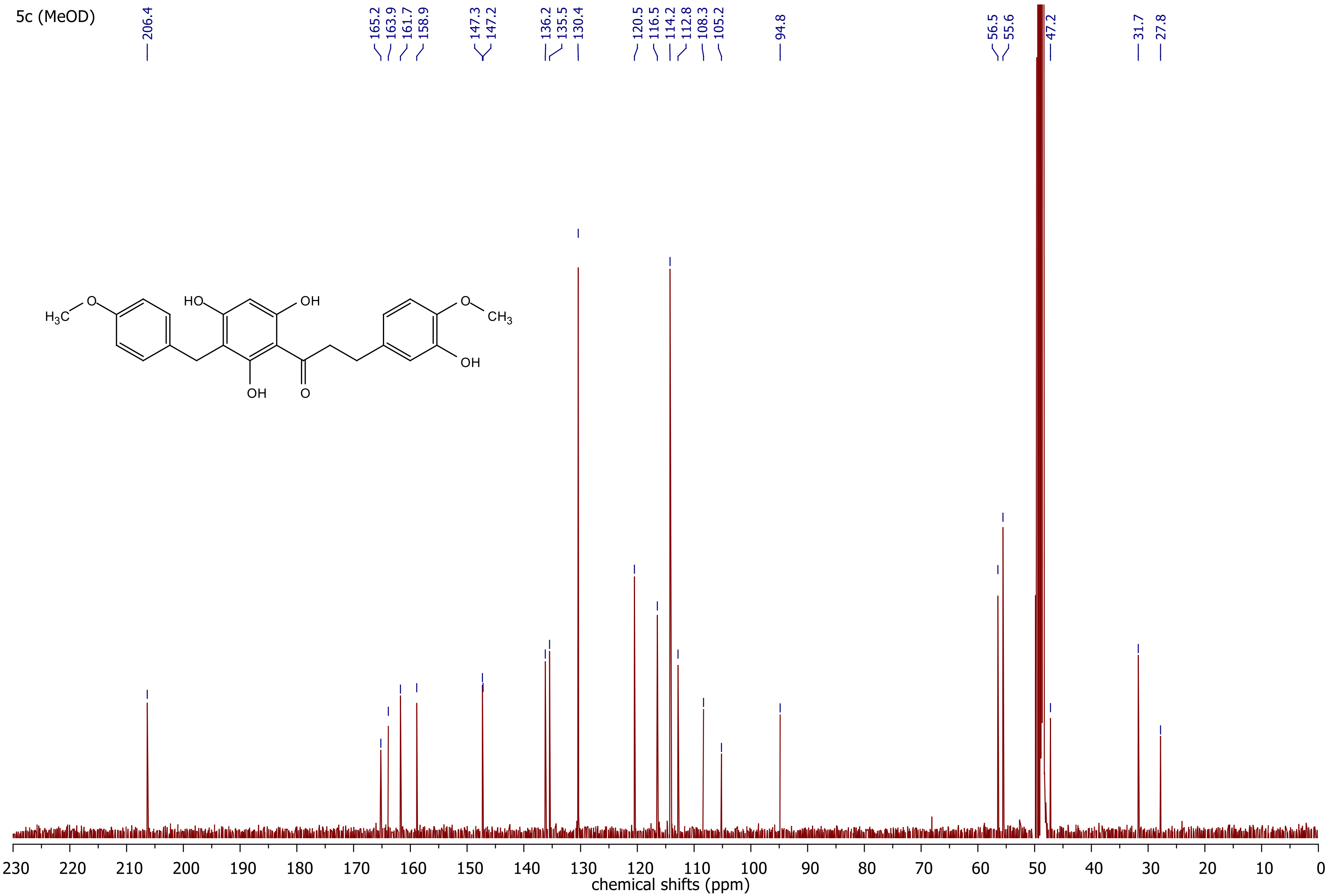

10. ^{1}H and ^{13}C NMR Spectra of compound **5d**

a. *Figure S19: ^{1}H NMR spectrum of Compound **5d***

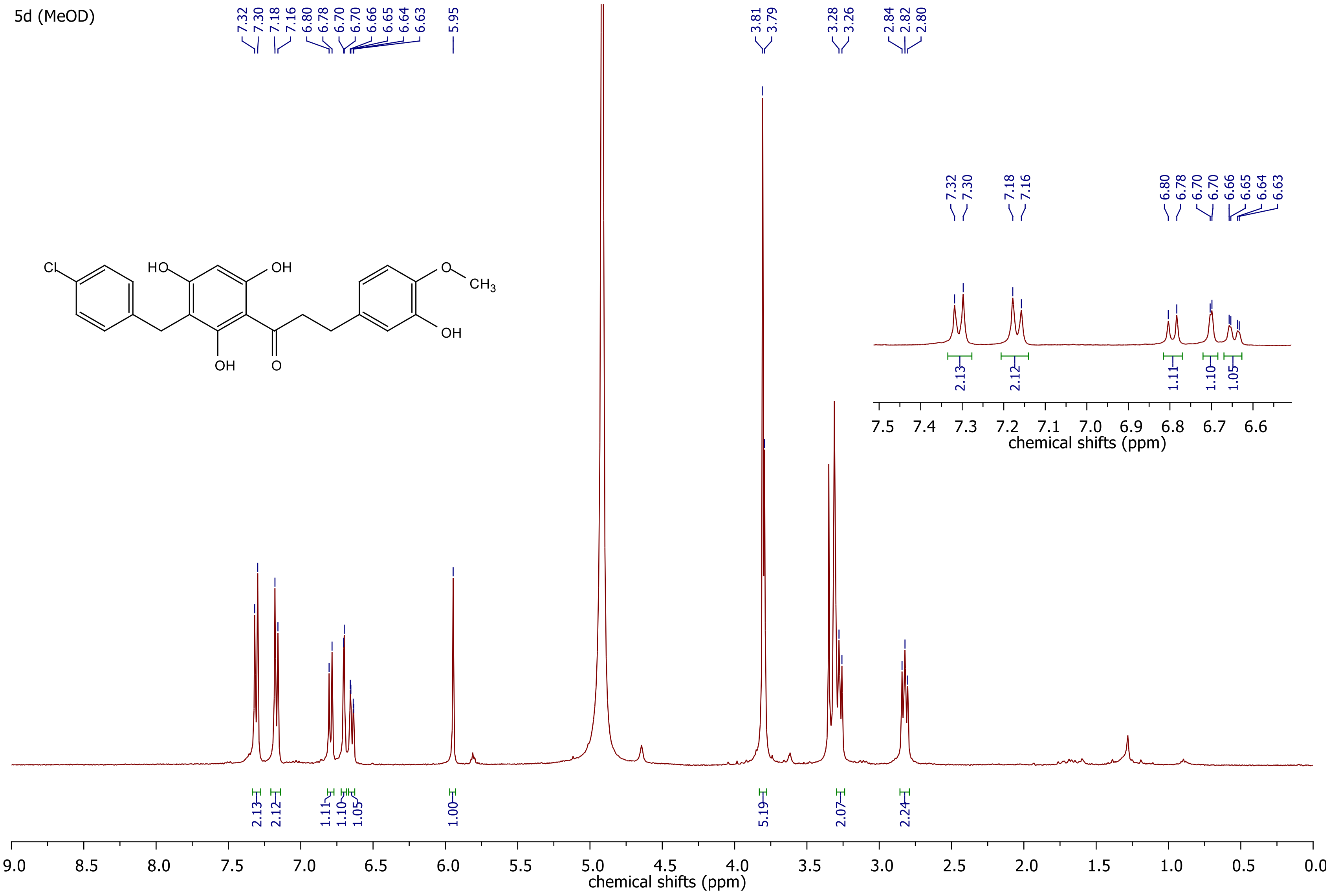

b. *Figure S20: ^{13}C NMR spectrum of Compound **5d***

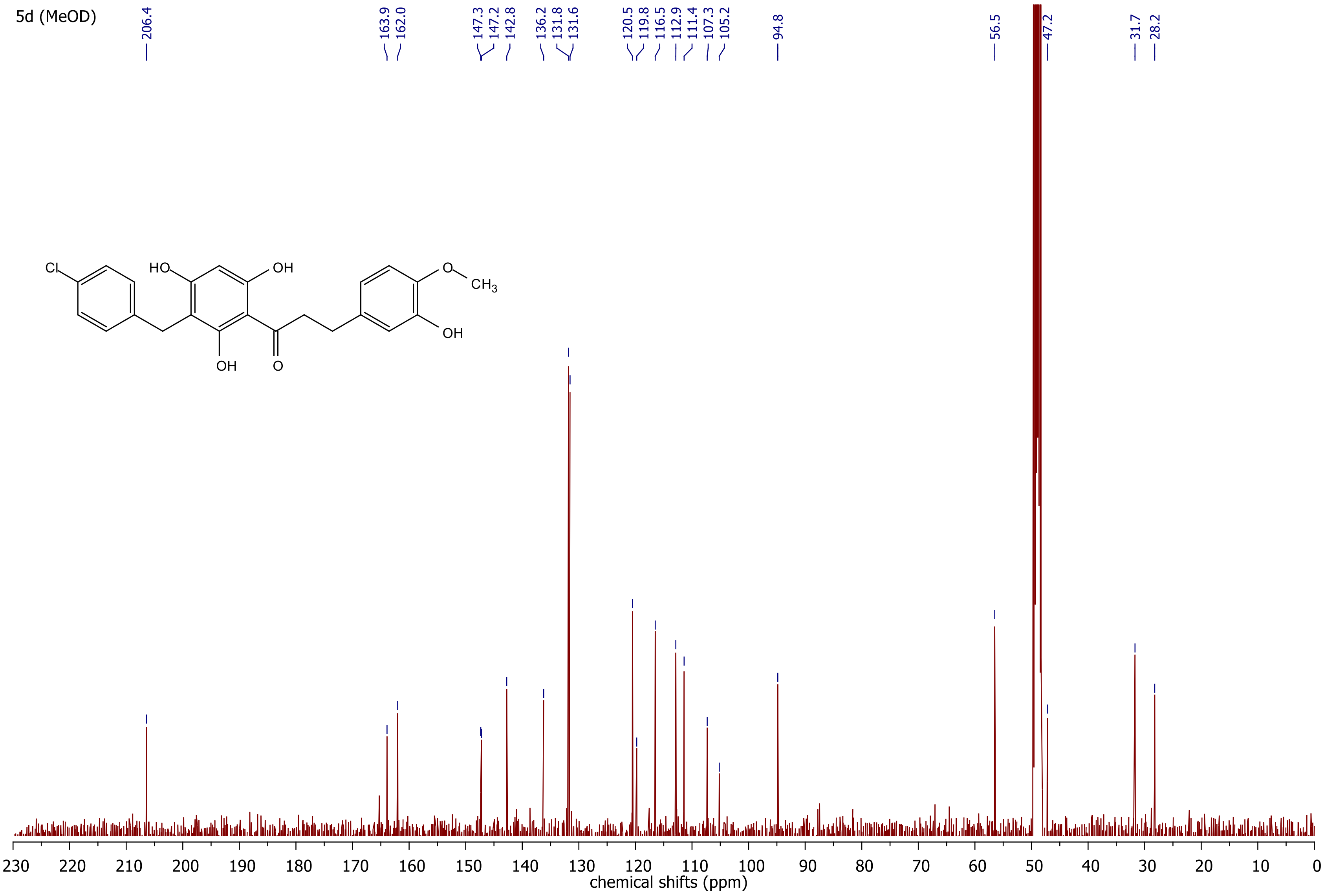

11. ¹H and ¹³C NMR Spectra of compound **7a**

a. Figure S21: ¹H NMR spectrum of Compound **7a**

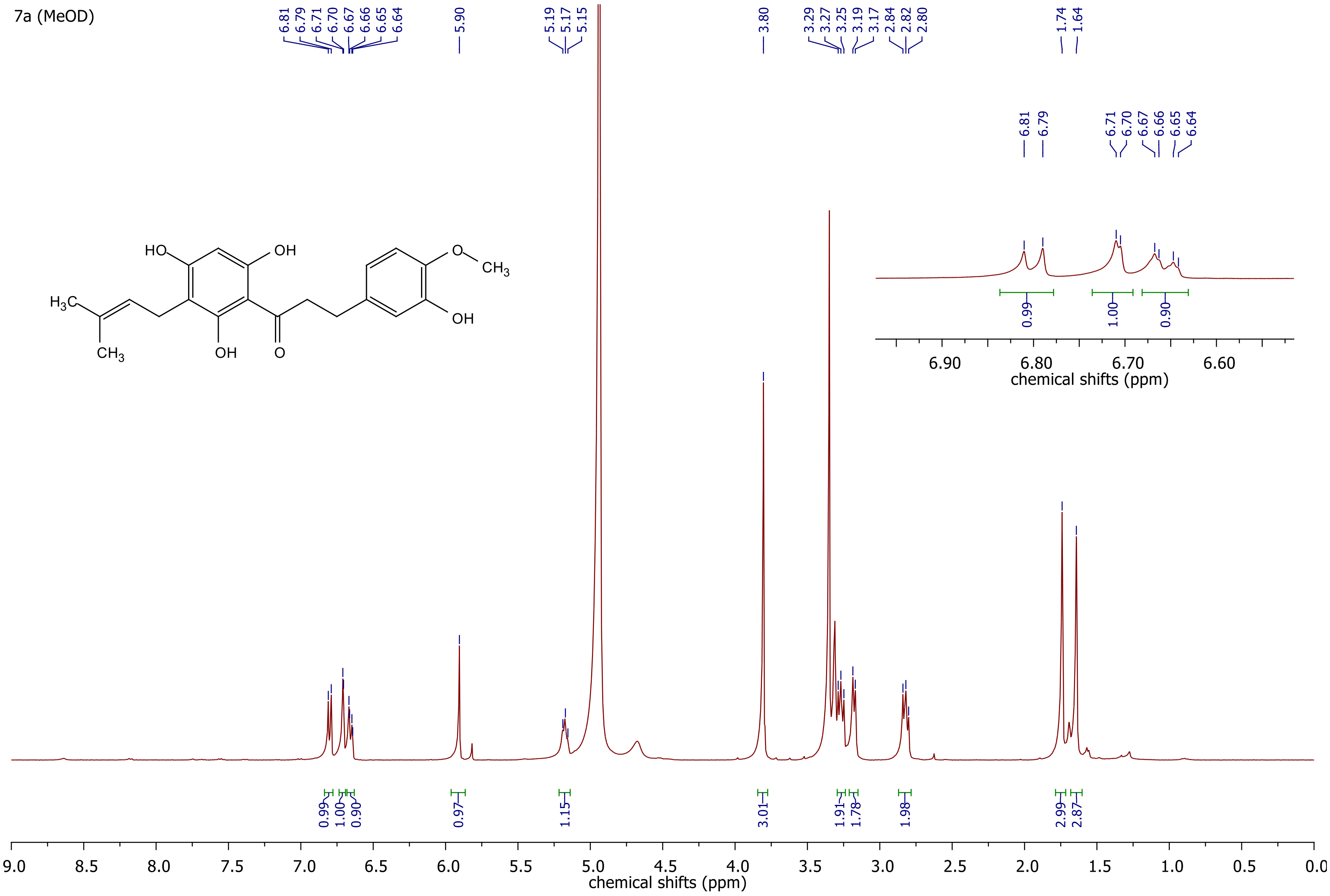

b. *Figure S22: ^{13}C NMR spectrum of Compound* ***7a***

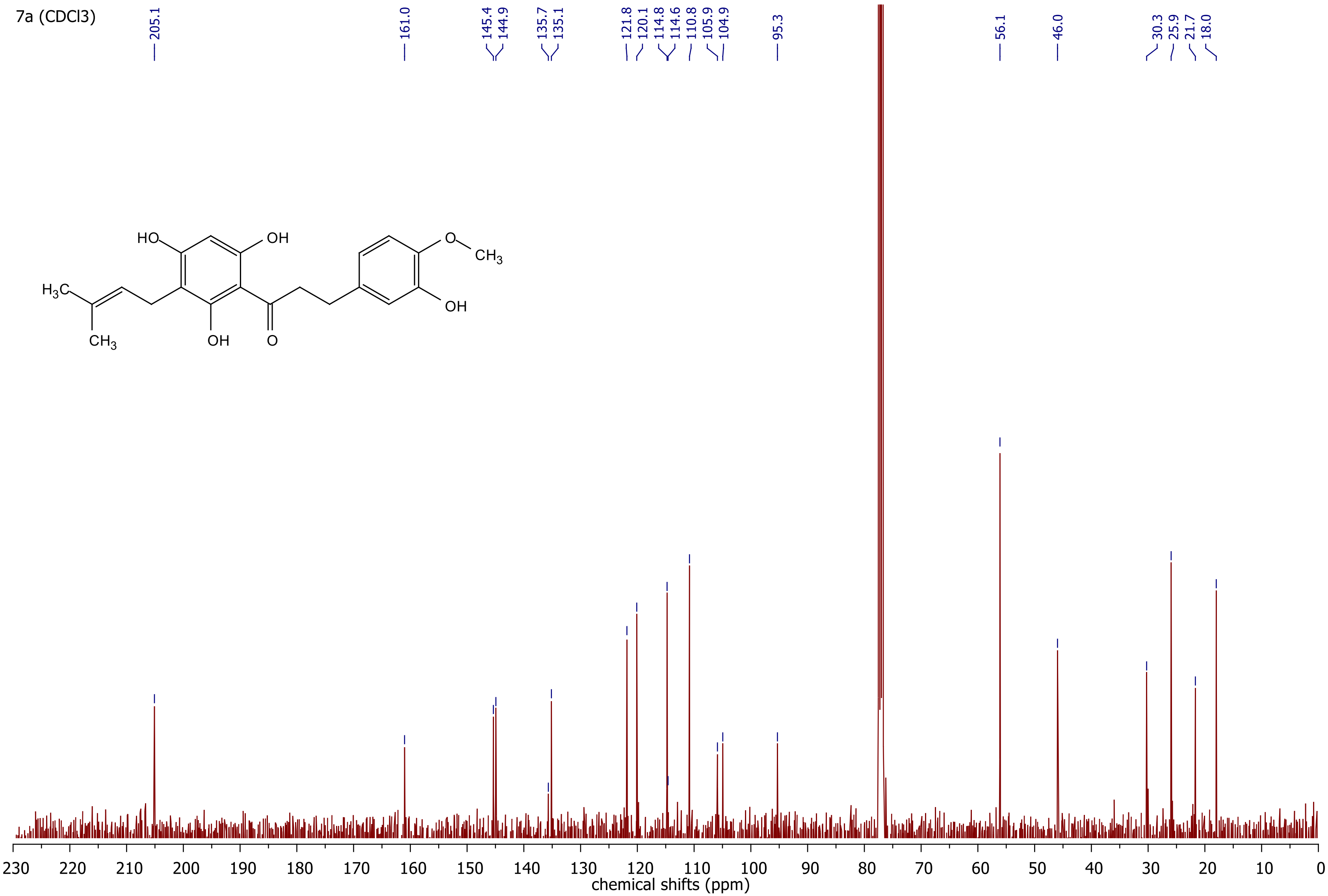

12. *^{1}H and ^{13}C NMR Spectra of compound* ***7b***

a. *Figure S23: ^{1}H NMR spectrum of Compound* ***7b***

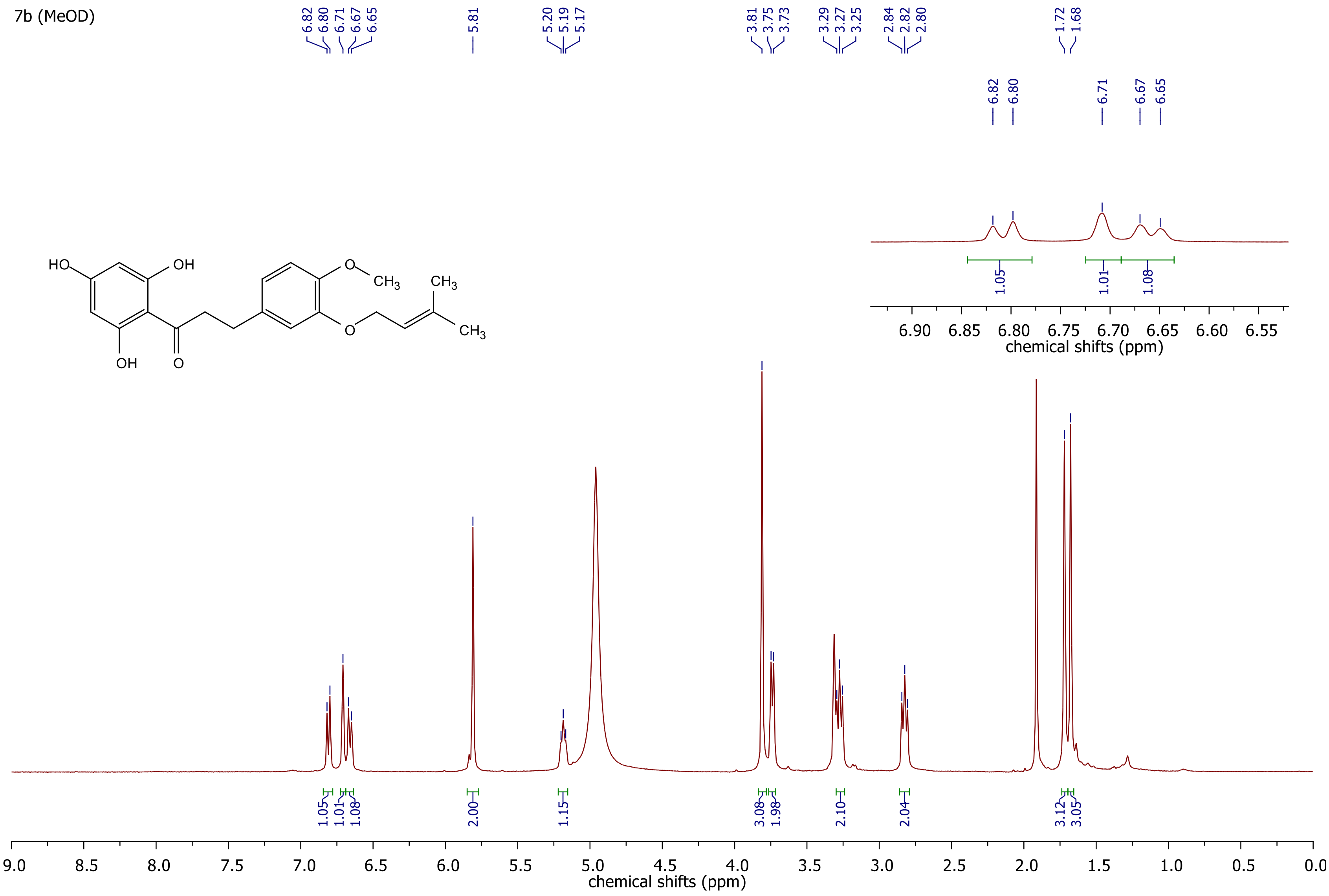

b. *Figure S24: ^{13}C NMR spectrum of Compound* ***7b***

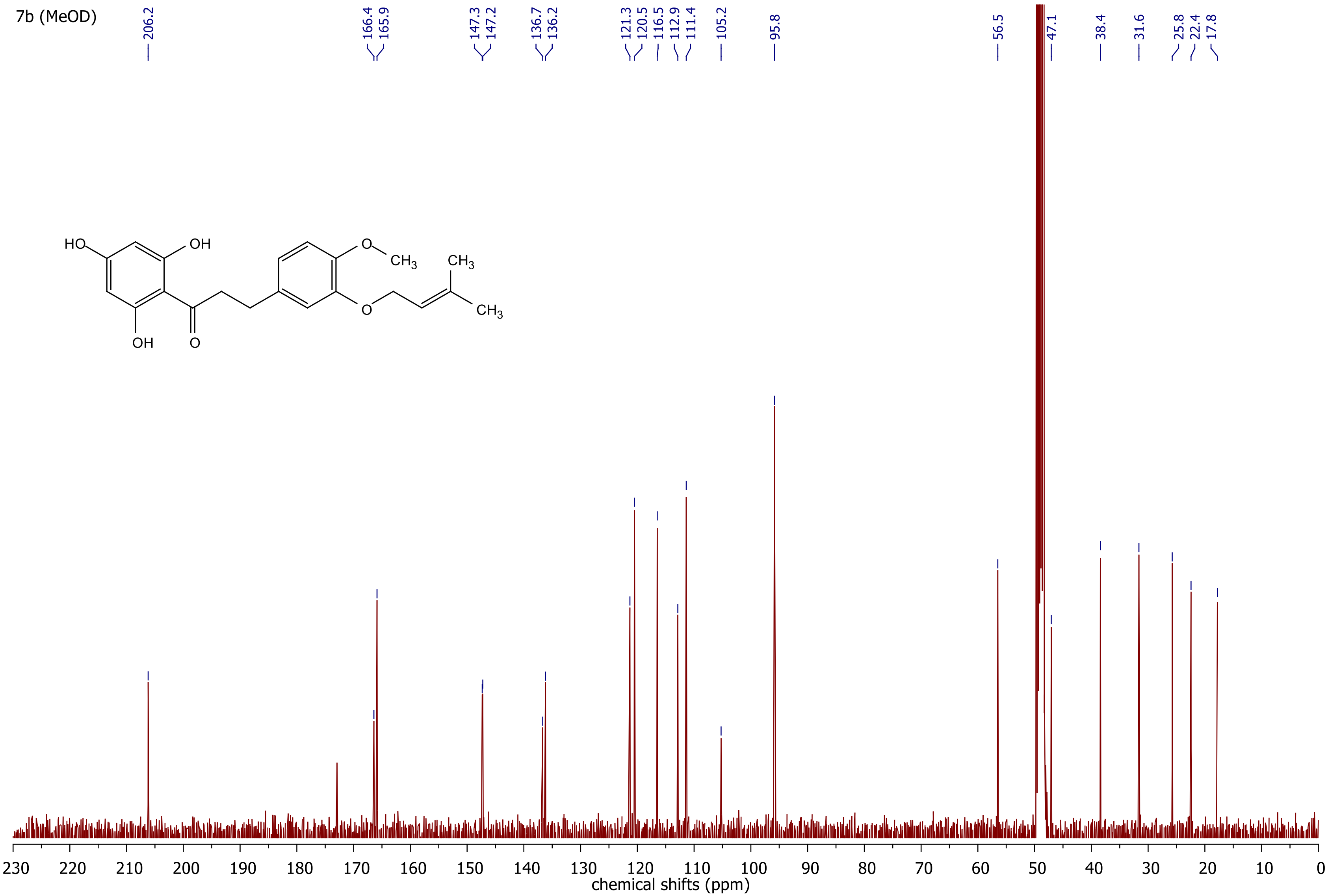

13. *^{1}H and ^{13}C NMR Spectra of compound **8a***

*a. Figure S25: ^{1}H NMR spectrum of Compound **8a***

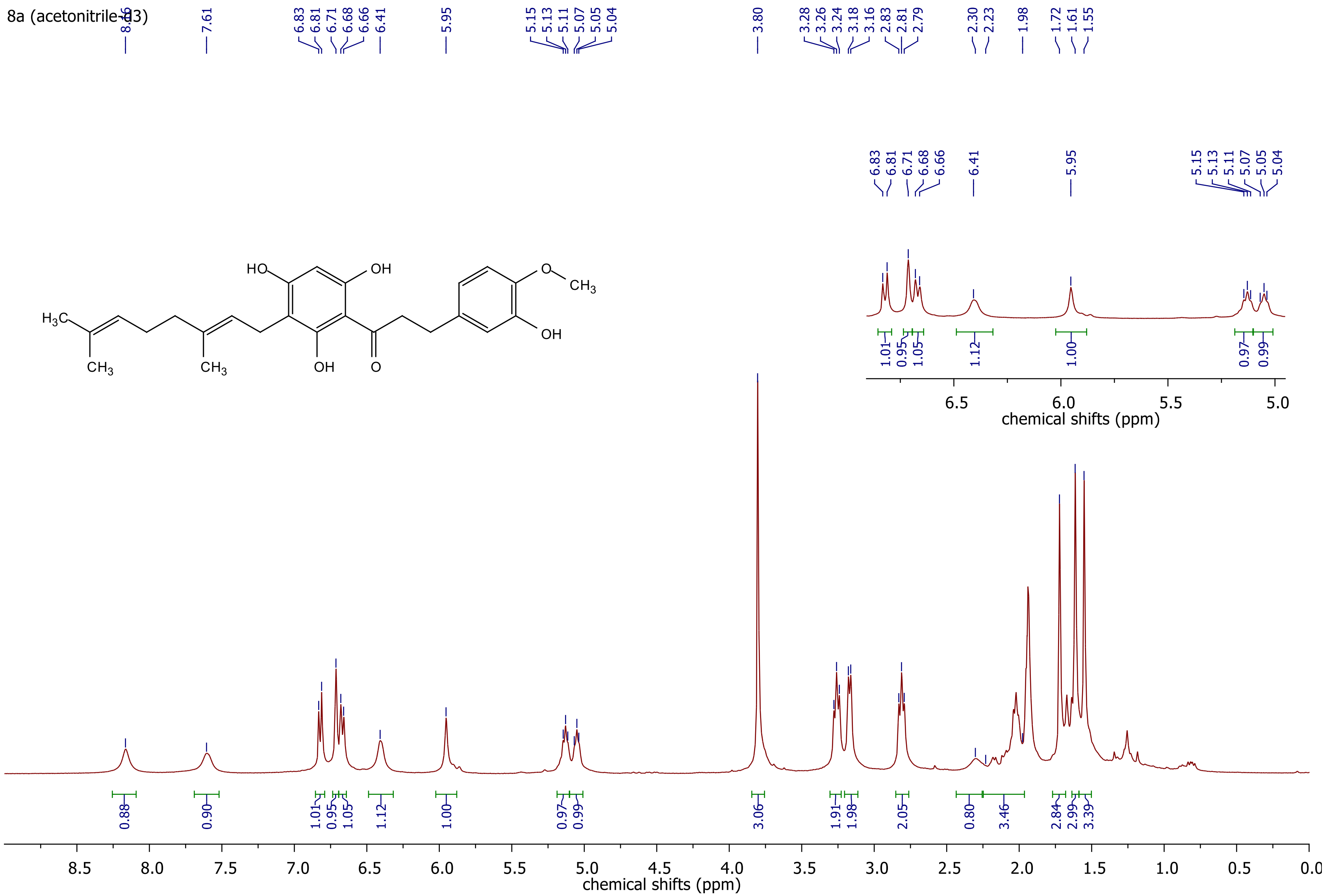

b. *Figure S26: ^{13}C NMR spectrum of Compound **8a***

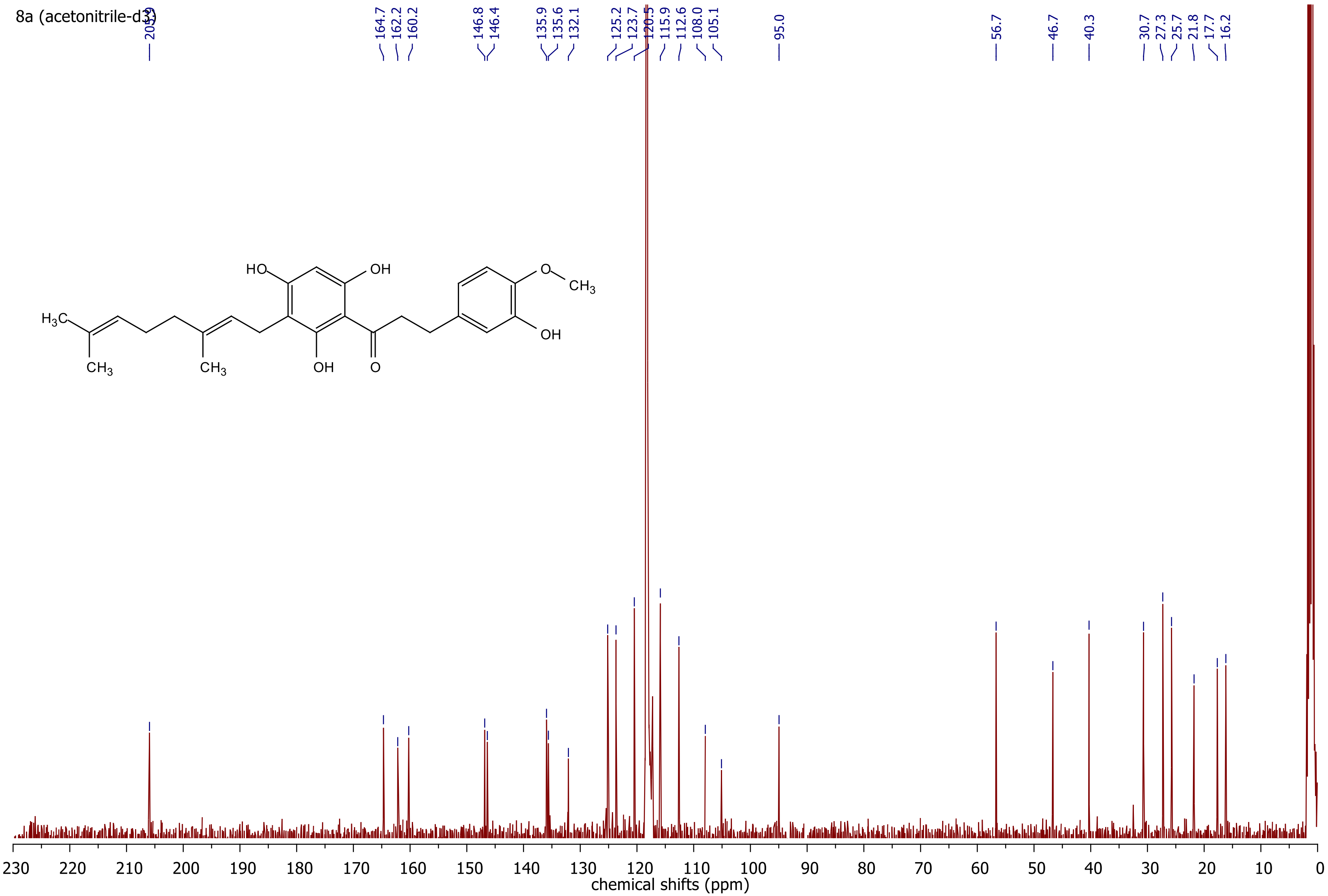

14. ^{1}H and ^{13}C NMR Spectra of compound **8b**

a. Figure S27: ^{1}H NMR spectrum of Compound **8b**

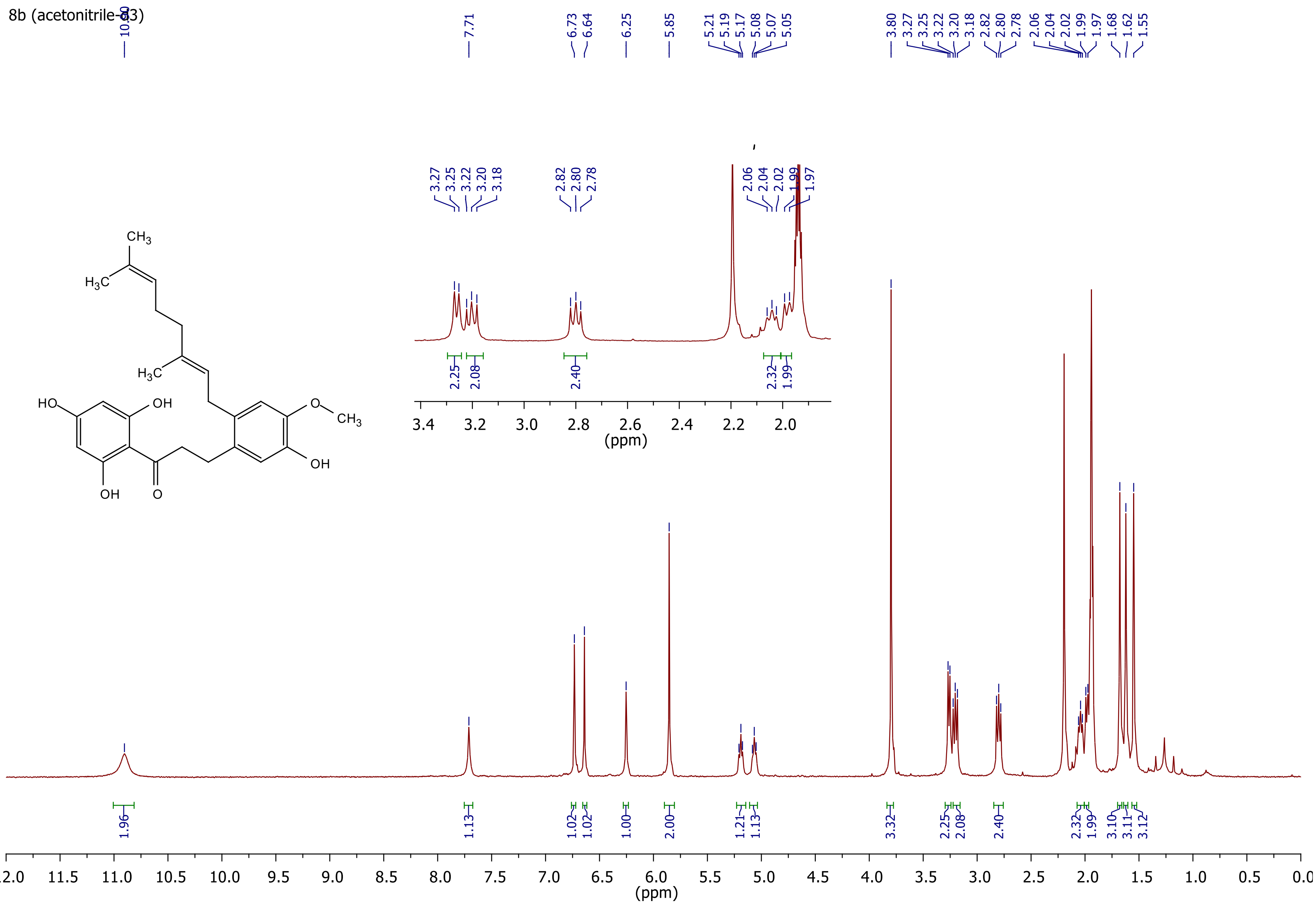

b. *Figure S28: ^{13}C NMR spectrum of Compound* ***8b***

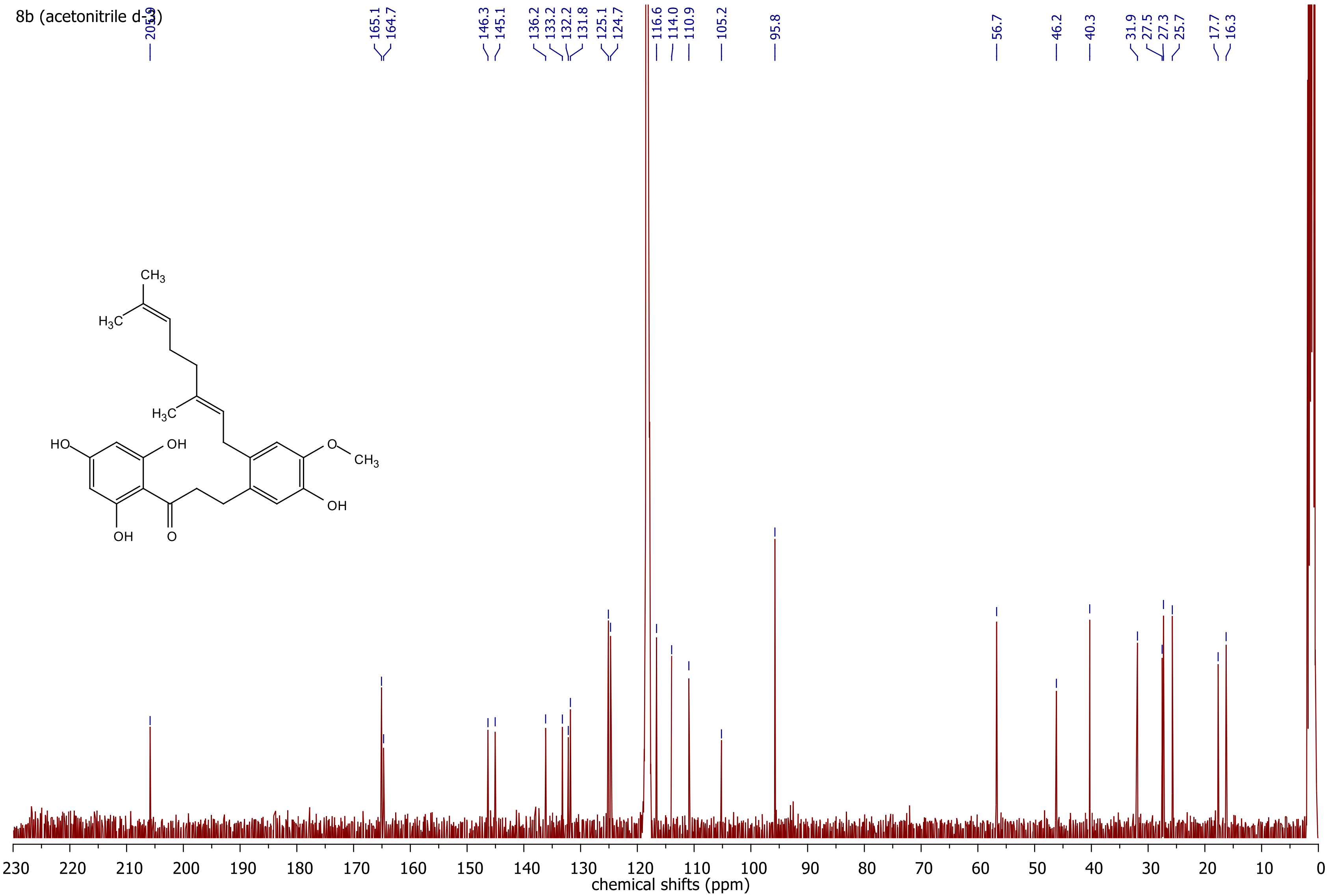

Supplement: Supplementary file 1 [file antibiotics-10-00620-s001.zip › antibiotics-1229060-supplementary.pdf]
